# Supplementary material for: Sex Differences in the Blood Transcriptome Identify Robust Changes in Immune Cell Proportions with Aging and Influenza Infection
Source: Cell Rep. 2019 Nov 12;29(7):1961–1973.e4. doi: 10.1016/j.celrep.2019.10.019 (PMC6856718; doi:10.1016/j.celrep.2019.10.019)
Supplement: Document S1. Figures S1–S6 [file mmc1.pdf]

Cell Reports, Volume *29*

Supplemental Information

Sex Differences in the Blood Transcriptome Identify Robust Changes in Immune Cell Proportions with Aging and Influenza Infection

Erika Bongen, Haley Lucian, Avani Khatri, Gabriela K. Fragiadakis, Zachary B. Bjornson, Garry P. Nolan, Paul J. Utz, and Purvesh Khatri

Supplemental Information
Contents
Fig. S1. Performance of XY- and Autosomal-iSEXS scores in discovery cohorts

Fig. S2. XXY-males express iSEXS scores and genes similarly to females

Fig. S3. Autosomal-iSEXS score reflects age-dependent sex differences in monocyte percentages.

Fig. S4. XY-iSEXS score does not change over time in females or males following influenza challenge

Fig. S5. iSEXS and Antibody Responses

Fig. S6. XY-iSEXS and Autosomal-iSEXS in SLE datasets

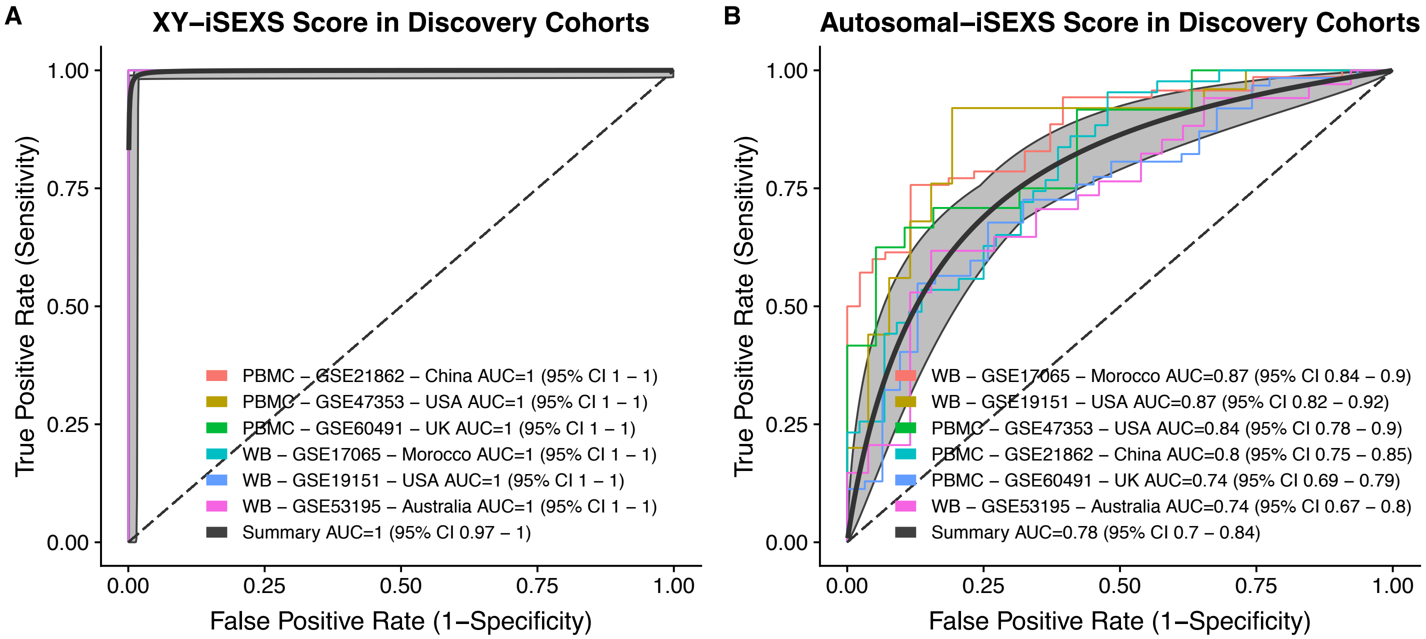

Figure S1. Performance of XY- and Autosomal-iSEXS scores in discovery cohorts. Related to Figure 2. ROC plots of the A) XY-iSEXS score (summary AUROC=1.00 95% CI 0.97-1.00) and B) Autosomal-iSEXS score (summary AUROC=0.78, 95% CI 0.7-0.84) performance in separating males and females 18-40 years old in the discovery cohorts.

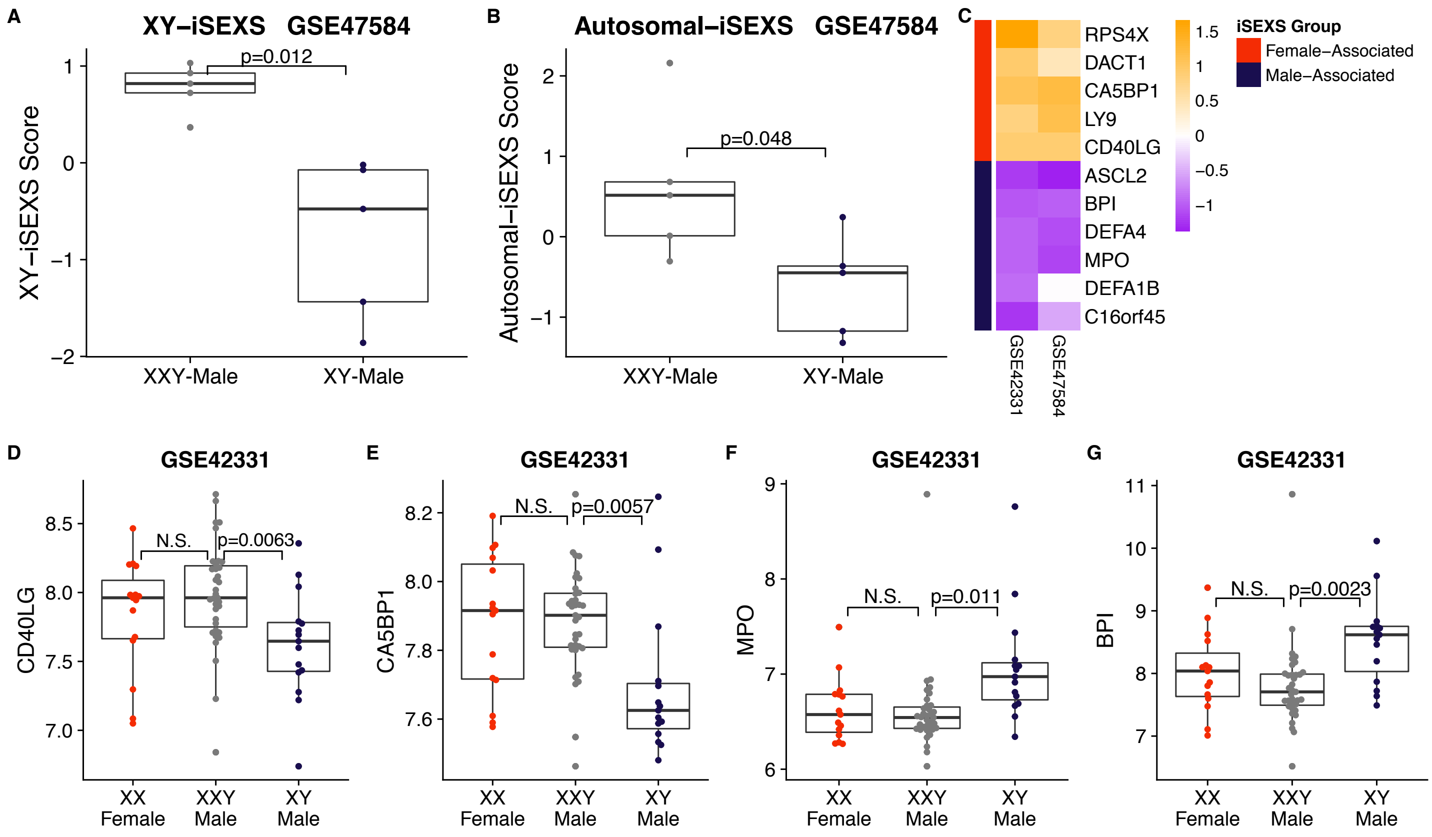

Figure S2. XXY-males express iSEXS scores and genes similarly to females. Related to Figure 2. Boxplots of A) XY-iSEXS and B) Autosomal-iSEXS scores in Klinefelter males (XXY-males) and typical males (XY-males). C) Heatmap of iSEXS genes significantly different between XXY-males and XY-males in a meta-analysis of GSE42331 and GSE47584 (FDR < 5%). Orange indicates genes higher expressed in XXY-males, purple indicates genes higher expressed in XY-males. Row color key indicates iSEXS genes higher expressed in females (red) or males (blue). Box plots of genes differentially expressed between XXY- and XY-males in cohort GSE42331: D) *CD40LG*, E) *CA5BP1*, F) *MPO*, and G) *BPI*.

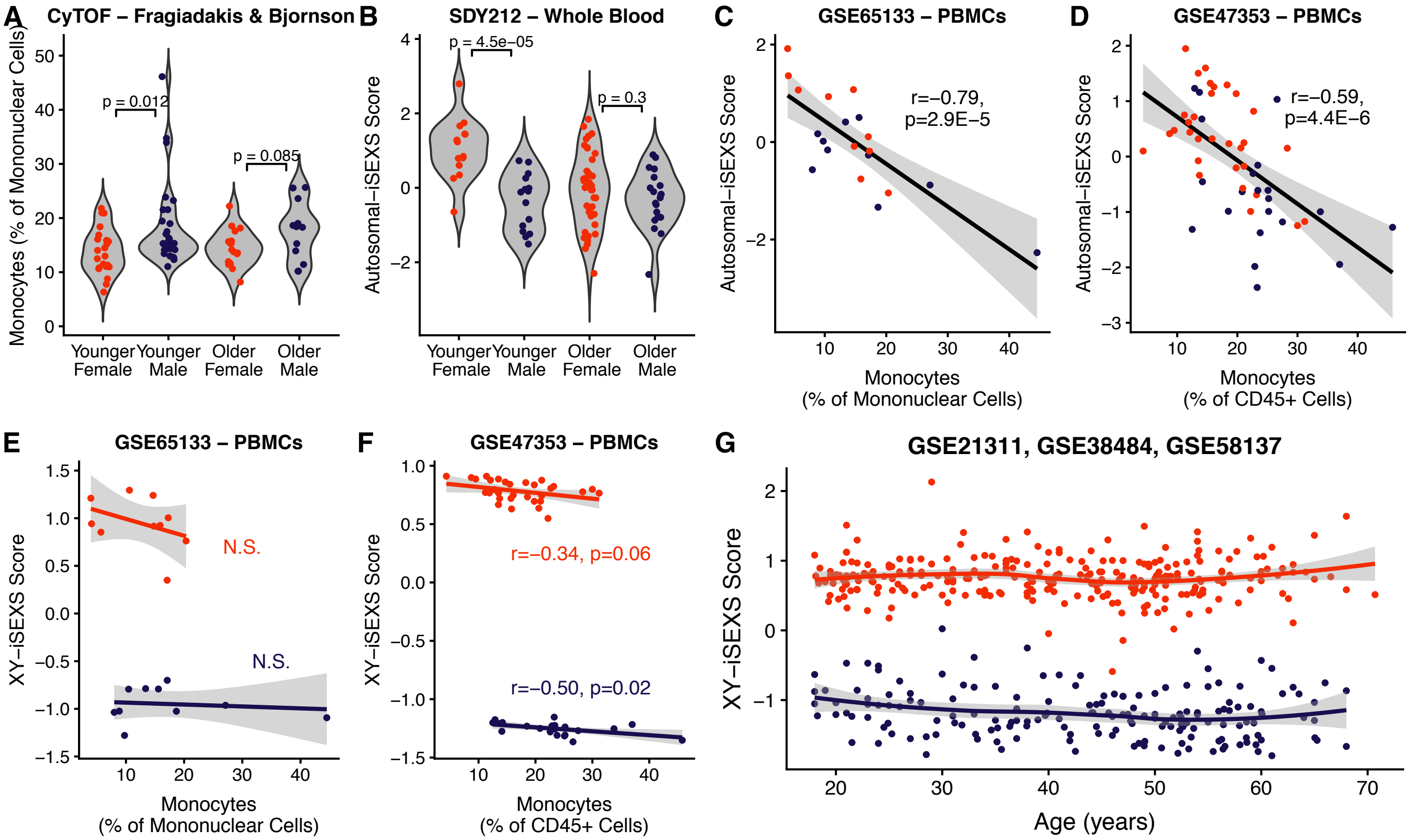

Figure S3. Autosomal-iSEXS score reflects age-dependent sex differences in monocyte percentages. Related to Figure 4. A) Among younger individuals (18-40 years old), males have a higher percentage of monocytes in the blood, as measured by CyTOF (p=0.012), but this difference is not significant in older individuals (41-63 years old; p=0.085). B) In SDY212, the Autosomal-iSEXS score is significantly higher in younger females (20-30 years old; p=4.5E-5), but demonstrates no significant difference in older individuals (60-90 years old; p=0.30). Autosomal-iSEXS scores correlate with flow cytometry measured monocyte percentages in C) GSE65133 (r=-0.79, p=2.9E-05) and D) GSE47353 (r=-0.59, p=4.4E-06). E) XY-iSEXS scores do not correlate with monocyte percentages GSE65133, (F) but significantly correlate in males in GSE47353 (r=-0.50, p=0.02) with a non-significant trend in females (r=-0.34, p=0.06). G) The XY-iSEXS scores are consistent across the life course in the combined cohorts of GSE21311, GSE38484, and GSE58137.

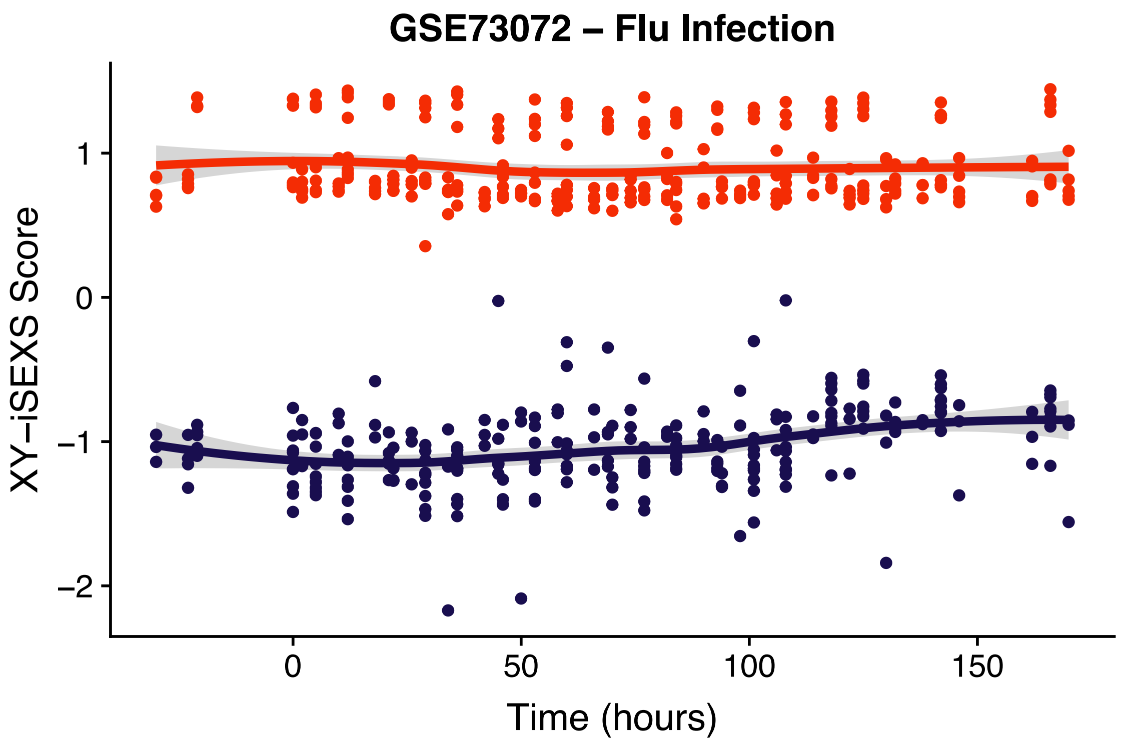

Figure S4. XY-iSEXS score does not change over time in females or males following influenza challenge. Related to Figure 5.

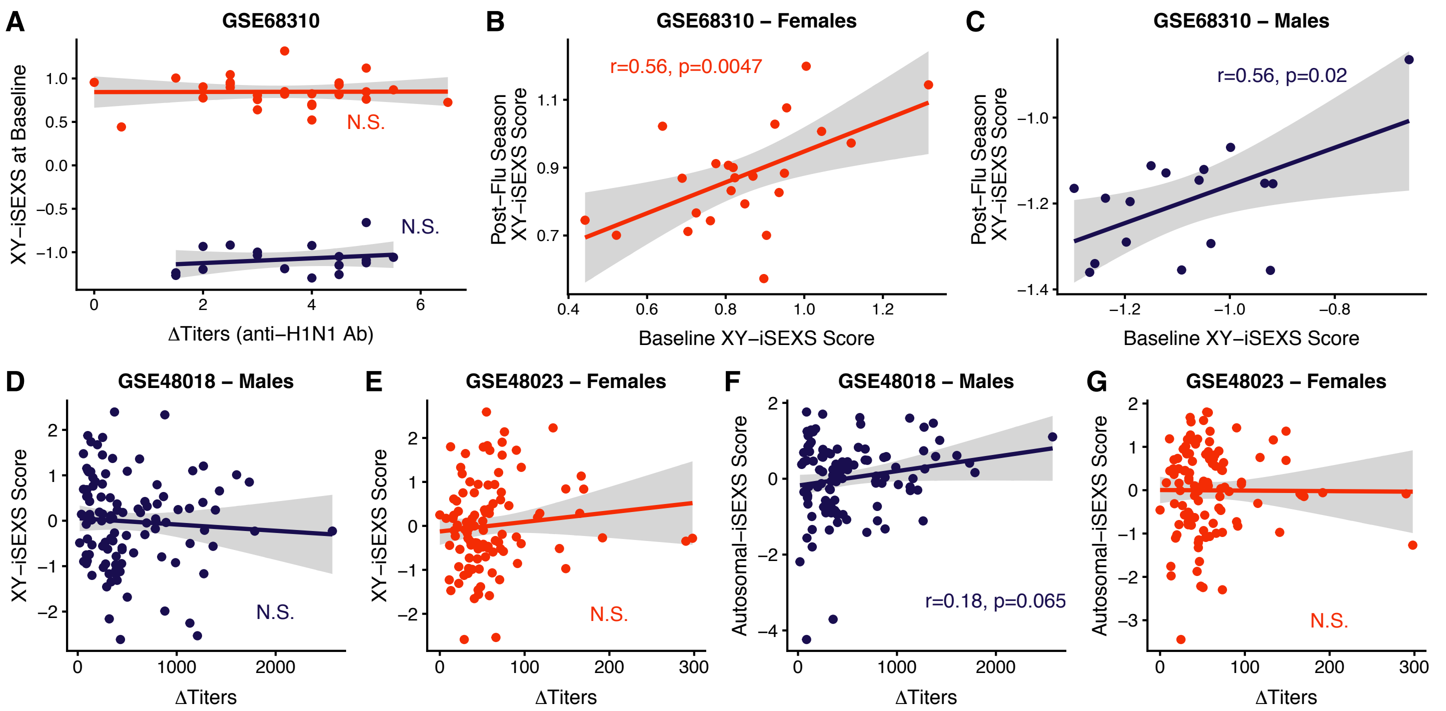

Figure S5. iSEXS and Antibody Responses. Related to Figure 6. A) XY-iSEXS at baseline is not correlated with change in titer post-influenza infection. B-C) XY-iSEXS score pre- and post-influenza infection is significantly correlated in females and males. Baseline D-E) XY-iSEXS score does not significantly correlate with antibody response to influenza vaccination. Correlation between baseline Autosomal-iSEXS score and ΔTiters in F) males (GSE48018; r=0.18, p=0.065) and G) females (GSE48023; r=-0.0063, p=0.95). In GSE48018 and GSE48023, antibody titers were calculated as the geometric mean of the antibody titers against the three influenza strains tested, and ΔTiters was the difference between the geometric mean titer at day 28 and day 0.

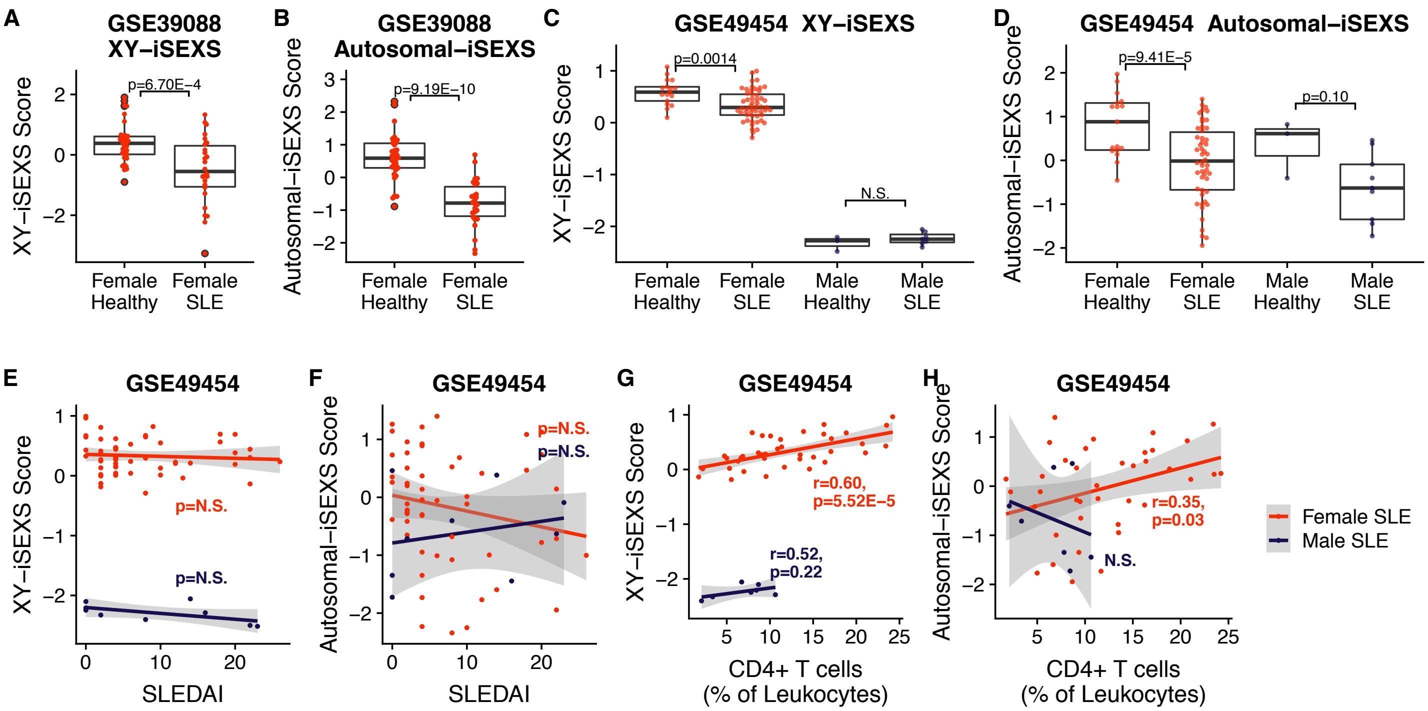

Fig. S6. XY-iSEXS and Autosomal-iSEXS in SLE datasets. Related to Figure 6. (A-D) XY-iSEXS and Autosomal-iSEXS are lower in females with SLE compared to healthy females. In males, Autosomal-iSEXS is significantly lower in males with SLE compared to healthy males, but XY-iSEXS is not. **(E-F)** XY-iSEXS is inversely correlated with SLEDAI in males but not in females. Autosomal-iSEXS is not correlated with SLEDAI in either females or males with SLE. **(G-H)** XY-iSEXS and Autosomal-iSEXS are positively correlated with proportion of CD4+ T cells in females, whereas only XY-iSEXS is negatively correlated in males. Autosomal-iSEXS is not correlated with proportions of CD4+ T cells in males.
