# Supplementary material for: Sex Differences in the Blood Transcriptome Identify Robust Changes in Immune Cell Proportions with Aging and Influenza Infection
Source: Cell Rep. 2019 Nov 12;29(7):1961–1973.e4. doi: 10.1016/j.celrep.2019.10.019 (PMC6856718; doi:10.1016/j.celrep.2019.10.019)
Supplement: Document S2. Article plus Supplemental Information [file mmc3.pdf]

Cell Reports

Article

Sex Differences in the Blood Transcriptome Identify Robust Changes in Immune Cell Proportions with Aging and Influenza Infection

Graphical Abstract

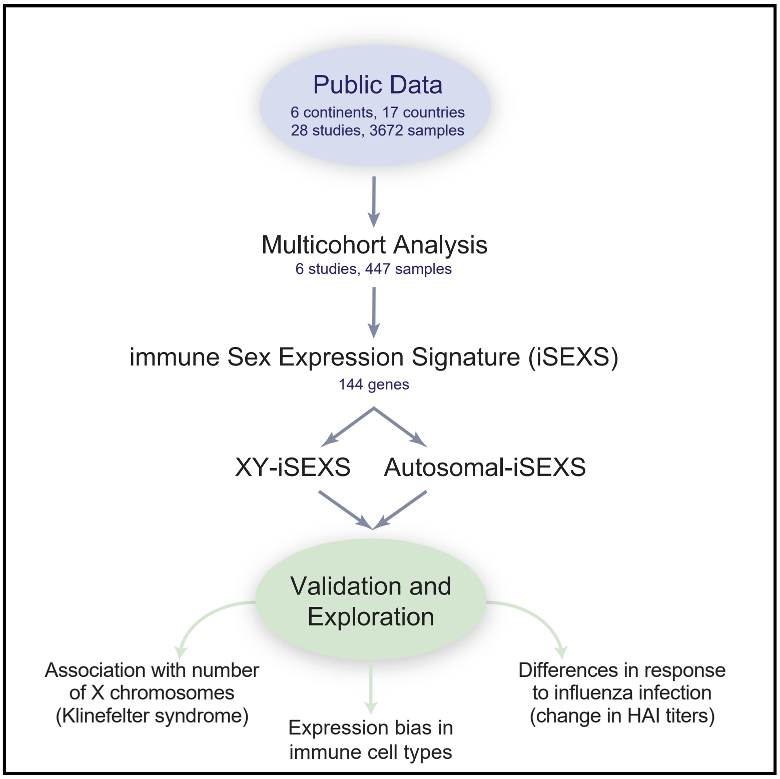

Highlights

- iSEXS is a gene signature capturing sex differences in healthy adult human blood
- Autosomal-iSEXS genes are driven by sex differences in immune cell proportions
- Autosomal-iSEXS reflects sex differences in influenza infection T cell dynamics
- Autosomal-iSEXS predicts antibody response to influenza in males, but not females

Authors

Erika Bongen, Haley Lucian, Avani Khatri, ..., Garry P. Nolan, Paul J. Utz, Purvesh Khatri

Correspondence

pjutz@stanford.edu (P.J.U.), pkhatri@stanford.edu (P.K.)

In Brief

Bongen et al. identify a 144-gene immune sex expression signature (iSEXS) that is differentially expressed in the blood of healthy human males and females. Many of the autosomal-iSEXS genes are driven by sex differences in immune cell proportions and predict antibody responses to influenza infection in males, but not females.

Bongen et al., 2019, Cell Reports *29*, 1961–1973
November 12, 2019 © 2019 The Authors.
https://doi.org/10.1016/j.celrep.2019.10.019

OPEN ACCESS
CellPress

Sex Differences in the Blood Transcriptome Identify Robust Changes in Immune Cell Proportions with Aging and Influenza Infection

Erika Bongen,[1,2] Haley Lucian,[1,5] Avani Khatri,[1,5] Gabriela K. Fragiadakis,[3,4] Zachary B. Bjornson,[3,4] Garry P. Nolan,[3,4] Paul J. Utz,[1,5,*] and Purvesh Khatri[1,6,7,*]
[1]Institute for Immunity, Transplantation and Infection, Stanford University School of Medicine, Stanford, CA 94305, USA
[2]Program in Immunology, Stanford University School of Medicine, Stanford, CA, USA
[3]Department of Microbiology & Immunology, Stanford University School of Medicine, Stanford, CA 94305, USA
[4]Baxter Laboratory for Stem Cell Biology, Stanford University School of Medicine, Stanford, CA 94305, USA
[5]Department of Medicine, Division of Immunology and Rheumatology, Stanford University School of Medicine, Stanford, CA 94305, USA
[6]Department of Medicine, Division of Biomedical Informatics Research, Stanford University School of Medicine, Stanford, CA 94305, USA
[7]Lead Contact
*Correspondence: pjutz@stanford.edu (P.J.U.), pkhatri@stanford.edu (P.K.)
https://doi.org/10.1016/j.celrep.2019.10.019

SUMMARY

Sex differences in autoimmunity and infection suggest that a better understanding of molecular sex differences will improve the diagnosis and treatment of immune-related disease. We identified 144 differentially expressed genes, referred to as immune sex expression signature (iSEXS), between human males and females using an integrated multi-cohort analysis of blood transcriptome profiles from six discovery cohorts from five continents with 458 healthy individuals. We validated iSEXS in 11 additional cohorts of 524 peripheral blood samples. When we separated iSEXS into genes located on sex chromosomes (XY-iSEXS) or autosomes (autosomal-iSEXS), both modules distinguished males and females. iSEXS reflects sex differences in immune cell proportions, with female-associated genes showing higher expression by CD4^{+} T cells and male-associated genes showing higher expression by myeloid cells. Autosomal-iSEXS detected an increase in monocytes with age in females, reflected sex-differential immune cell dynamics during influenza infection, and predicted antibody response in males, but not females.

INTRODUCTION

Sex differences in immune responses lead to different risks for immunological diseases (Pennell et al., 2012). Women have a greater risk for autoimmune diseases such as systemic lupus erythematosus (SLE) and systemic sclerosis (SSc), which both affect nine women per one man (Libert et al., 2010). In contrast, men are more likely to die of infectious and parasitic disease in the United States, particularly between 25 and 65 years of age (Owens, 2002), and have a greater risk of non-reproductive cancers (Cook et al., 2011; Dorak and Karpuzoglu, 2012; Klein and Flanagan, 2016). Differences between male and female immune responses are mediated by both gender and sex. Gender encompasses the behaviors that a given society defines as masculine or feminine, whereas sex encompasses biological factors that differ between males and females, including hormonal milieu and chromosome complement (Klein and Flanagan, 2016). Estrogens and testosterones are thought to be pro-inflammatory and anti-inflammatory, respectively (Furman et al., 2014). Female-like risk for SLE in XXY males with Klinefelter syndrome suggests that X chromosome dosage plays a role in autoimmunity risk (Souyris et al., 2018). Studies in SLE mouse models indicate that both hormonal milieu and chromosome complement play roles in disease risks, as XY-female mice have less severe SLE-like disease than XX-female mice (Sasidhar et al., 2012). Many studies have reported transcriptional sex differences in peripheral blood (Eady et al., 2005; Jansen et al., 2014; Melé et al., 2015; Whitney et al., 2003; Toker et al., 2016), but few have investigated transcriptional sex differences within the context of immune responses in healthy adults (Liang et al., 2017; Piasecka et al., 2018). Importantly, virtually every study to date used only one cohort, mostly consisting of individuals of European descent, without demonstrating robustness across independent cohorts. Further, using a single homogeneous cohort makes it difficult to segregate effects of sex and gender or an interaction between the two in immune response.

We hypothesized that robust differences in the immune systems of healthy men and women exist across worldwide populations. We further hypothesized that using multiple cohorts from different continents would minimize society-specific gender differences and illuminate biologically driven sex differences in the immune system. To test these hypotheses, we analyzed publicly available blood transcriptome data from geographically diverse independent cohorts of men and women aged 18–40 years to identify a transcriptional signature of sex difference called the immune sex expression signature (iSEXS). After validating iSEXS in 11 independent cohorts, we divided iSEXS genes by chromosomal location into XY-iSEXS and autosomal-iSEXS. We investigated iSEXS in 16 cohorts to identify immune cells that drive iSEXS, determine the effect of aging on iSEXS, and understand

This is an open access article under the CC BY license (http://creativecommons.org/licenses/by/4.0/).

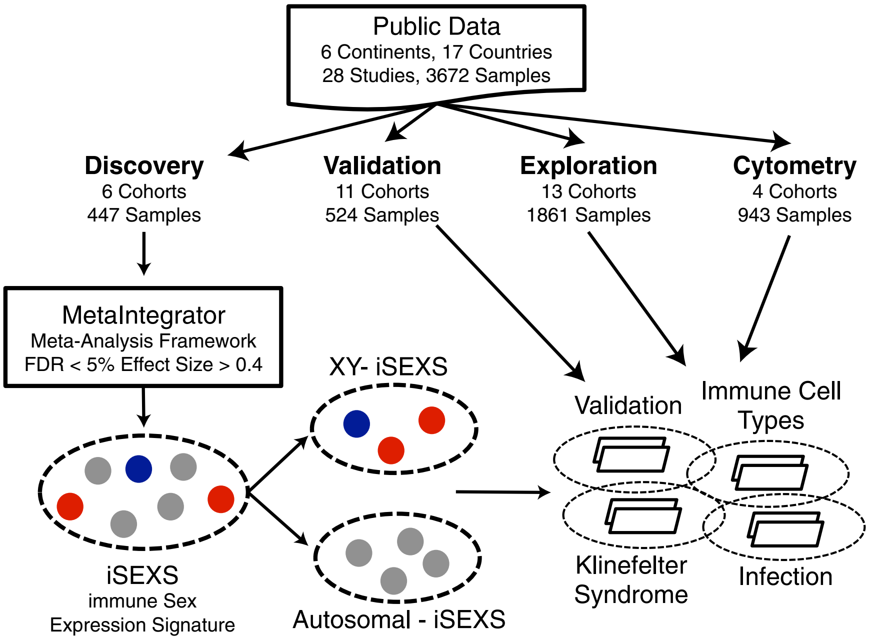

Figure 1. Identification, Validation, and Exploration of the Immune Sex Expression Signature (iSEXS)

We downloaded publicly available gene expression microarray and flow cytometry datasets composed of 28 independent studies with 3,672 samples from 17 countries and 6 continents. We performed a meta-analysis of 6 Discovery cohorts with 447 samples to identify genes differentially expressed in the blood between healthy males and females 18–40 years old. We identified iSEXS as the genes with FDR <5% and |effect size| >0.4. We separated iSEXS based on chromosomal location into autosomal-iSEXS and XY-iSEXS. We tested autosomal-iSEXS and XY-iSEXS expression in 11 validation cohorts with 524 samples. Using 13 exploration cohorts with 1,861 samples, we examined autosomal-iSEXS and XY-iSEXS in Klinefelter syndrome, immune cells, and infection. We included some discovery and validation cohorts within exploration analyses.

the relationship between baseline iSEXS and antibody responses to influenza infection. We found that iSEXS captures robust sex differences in healthy humans from worldwide populations, irrespective of their genetic background, and has implications for how sex differences at baseline can affect sex differences in immune response outcomes.

RESULTS

Dataset Description

We downloaded 28 publicly available gene expression datasets consisting of 3,672 samples from the blood of human adults (Figure 1). Of these, we selected six transcriptome datasets of whole blood or peripheral blood mononuclear cells (PBMCs) from 447 healthy individuals (258 females, 189 males) aged 18–40 years (Table 1) from five continents to ensure genetic and cultural diversity within the discovery cohorts. For the validation cohorts, we selected 11 cohorts of 524 healthy individuals (263 females and 261 males) 18–40 years old from six continents (Table 1). We used 13 datasets of 1,861 samples to expand our analysis to individuals with Klinefelter syndrome, influenza infection, SLE, and older individuals who were not part of the discovery process, referred to as exploration cohorts (Table 1). We also downloaded transcriptome data for 279 healthy individuals (152 females and 127 males) from the Milieu Intérieur cohort (Piasecka et al., 2018) for validation (Table 1). Finally, we used four cytometry cohorts with 943 samples to investigate the association between the blood transcriptome and immune cell type proportions (Table 1).

Identification of a Robust Sex Difference Gene Signature

We identified 144 differentially expressed genes (false discovery rate [FDR] ≤5% and |effect size| ≥0.4) between females and males, referred to as iSEXS (Figure 2A; Table S1), of which 94 were higher expressed in females (female-associated genes) and 50 were higher expressed in males (male-associated genes). As expected, iSEXS was enriched for X chromosome (25 genes, p = 3.66e-11) and Y chromosome genes (9 genes, p = 3.39e-7). 17 out of the 25 genes on the X chromosome were known X-escape genes, which may be higher expressed in females due to expression from both copies of the X chromosome (Tukiainen et al., 2017). Two genes (*ZBED1* and *CD99*) expressed higher in males are located in pseudoautosomal region 1 (PAR1), a region of the Y chromosome that shares homology with the X chromosome (Libert et al., 2010). Importantly, 75% of iSEXS genes were located on autosomes, indicating that transcriptional sex differences are not limited to genes on sex chromosomes. Furthermore, 123 out of 144 genes showed no heterogeneity in the discovery cohorts; only 21 genes showed some amount of heterogeneity (Table S1).

We validated iSEXS in the 11 held-out validation cohorts (Table 1). Out of 144 genes in iSEXS, 130 genes showed the same direction of change, of which 80 were statistically significant ($p < 0.05$) (Figure 2B; Table S1). We created forest plots of the validation cohort effect sizes of *CD40LG* (chromosome X) and *CTSG* (chromosome 14; Figure 2C) to illustrate the consistency in expression of genes in iSEXS. Both genes demonstrate consistent effect sizes in datasets from Africa, Asia, Australia, Europe, and North and South America. Next, we validated a subset of the iSEXS genes in the Milieu Intérieur Consortium cohort, which is a population study of 1,000 healthy French individuals aged 20–70 years old (Piasecka et al., 2018). Because the Milieu Intérieur Consortium *a priori* selected which genes to profile using NanoString, only 13 iSEXS genes were measured. In the 279 individuals (152 females and 127 males) aged 20–40 years old in the Milieu Intérieur Consortium cohort, all but one of these 13 genes exhibited effect sizes in the same direction, of which 10 genes were statistically significant (p value < 0.05; Figure 2D).

Table 1. Discovery, Validation, Exploration, and Cytometry Cohorts

Dataset	Category	Reference	Country	Tissue	Manufacturer	Composition	Total
GSE17065	discovery	(Idaghdour et al., 2010)	Morocco	WB	Illumina	females: 70; males: 43	113
GSE19151	discovery	(Lewis et al., 2011)	USA	WB	Affymetrix	females: 25; males: 26	51
GSE21862	discovery	(McHale et al., 2011)	China	PBMC	Illumina	females: 43; males: 44	87
GSE47353	discovery	(Tsang et al., 2014)	USA	PBMC	Affymetrix	females: 24; males: 19	43
GSE53195	discovery	(Hemani et al., 2014)	Australia	WB	Illumina	females: 34; males: 26	60
GSE60491	discovery	(Vedhara et al., 2015)	UK	PBMC	Illumina	females: 62; males: 31	93
Total			5 countries			females: 258; males: 189	447
GSE13485	validation	(Querec et al., 2009)	USA	PBMC	Affymetrix	females: 13; males: 7	20
GSE18323	validation	(Vahey et al., 2010)	USA	PBMC	Affymetrix	females: 15; males: 33	48
GSE19442	validation	(Berry et al., 2010)	South Africa	WB	Illumina	females: 20; males: 11	31
GSE21311	validation	(Mason et al., 2010)	Australia	WB	Illumina	females: 21; males: 19	40
GSE30453	validation	(Heinzen et al., 2008)	USA	PBMC	Affymetrix	females: 12; males: 29	41
GSE37069	validation	(Seok et al., 2013)	USA	WB	Affymetrix	females: 11; males:18	29
GSE38484	validation	(de Jong et al., 2012)	Netherlands, Denmark	WB	Illumina	females: 30; males: 24	54
GSE58137	validation	(Peters et al., 2015)	USA	WB	Illumina	females: 75; males: 24	99
GSE61821	validation	(Hoang et al., 2014)	Singapore	WB	Illumina	females: 34; males: 80	114
GSE65219	validation	(Kuparinen et al., 2013)	Finland	PBMC	Illumina	females: 21; males: 9	30
GSE85263	validation	(Rojas-Pena et al., 2018)	Colombia	PBMC	Illumina HiSeq	females: 11; males: 7	18
Total			8 countries			females: 263; males: 261	524
GSE47584	exploration	(Huang et al., 2015)	China	WB	Agilent	XXY males: 5<br>XY males: 5	10
GSE42331	exploration	(Zitzmann et al., 2015)	Germany	WB	Affymetrix	XX females: 15<br>XY males: 15<br>XXY males: 35	65
GSE21311	exploration	(Mason et al., 2010)	Australia	WB	Illumina	females: 49<br>males: 48	97
GSE38484	exploration	(de Jong et al., 2012)	Netherlands, Denmark	WB	Illumina	females: 51<br>male: 39	90
GSE58137	exploration	(Peters et al., 2015)	USA	WB	Illumina	females: 165<br>males: 83	248
SDY212	exploration	(Furman et al., 2013)	USA	WB	Illumina	females: 53<br>males: 35	88
GSE73072	exploration	(Liu et al., 2016)	UK	WB	Affymetrix	females: 15<br>males: 14<br>time points: 20	542
GSE68310	exploration	(Zhai et al., 2015)	USA	WB	Illumina	females: 27<br>males: 18<br>time points: 2	86
GSE48018	exploration	(Bucasas et al., 2011)	USA	WB	Illumina	males: 108	108
GSE48023	exploration	(Bucasas et al., 2011)	USA	WB	Illumina	females: 106	106
GSE39088	exploration	(Lauwerys et al., 2013)	Belgium, Bulgaria, Croatia, France, Germany, Switzerland	WB	Affymetrix	SLE females: 26<br>control females: 34	60
GSE49454	exploration	(Chiche et al., 2014)	France	WB	Illumina	SLE females: 53<br>control females: 17<br>SLE males: 9<br>control males: 3	82

(*Continued on next page*)

Table 1. Continued

Dataset	Category	Reference	Country	Tissue	Manufacturer	Composition	Total
Milieu Intérieur	exploration	(Piasecka et al., 2018)	France	WB	NanoString	females: 152<br>males: 127	279
Total			12 countries			females: 763; males: 504; XXY males: 40	1,861
Milieu Intérieur	cytometry; exploration	(Patin et al., 2018)	France	WB	cytometry (flow)	females: 391; males: 389	780
Fragiadakis et al.	cytometry; exploration	Fragiadakis et al., 2019	USA	PBMC	CyTOF	females: 39; males: 44	83
GSE47353	cytometry; exploration	(Tsang et al., 2014)	USA	PBMC	flow cytometry	females: 37; males: 23	60
GSE65133	cytometry; exploration	(Newman et al., 2015)	USA	PBMC	flow cytometry	females: 10; males: 10	20
Total			2 countries			females: 477; males: 466	943

WB, whole blood.

Autosomal-iSEXS Score Distinguishes Males and Females

Next, we defined the XY-iSEXS and autosomal-iSEXS scores using genes located on sex chromosomes or autosomes, respectively. As expected, the XY-iSEXS scores distinguished males and females in discovery cohorts (summary area under the receiver operating characteristic curve (AUROC) = 1.00; 95% confidence interval [CI], 0.97-1.00; Figure S1A) and validation cohorts (summary area under the curve (AUC) = 0.99; 95% CI, 0.94-1.0; Figure 3A) with very high accuracy. The autosomal-iSEXS scores also distinguished males and females consistently, albeit with lower accuracy than XY-iSEXS scores in the discovery cohorts (summary AUROC = 0.78; 95% CI, 0.70-0.84; Figure S1B) and validation cohorts (summary AUC = 0.75, 95% CI 0.67-0.83, Figure 3B). These results further demonstrate that autosomal genes in iSEXS represent nuanced but robust sex differences.

X Chromosome Dosage Is Associated with Autosomal-iSEXS Score

Next, we investigated whether XY-iSEXS and autosomal-iSEXS scores were associated with the number of X chromosomes present in an individual subject. Males with Klinefelter syndrome have two X chromosomes (karyotype 47,XXY), which leads to increased estrogen and decreased testosterone levels (Groth et al., 2013). GSE42331 profiled XX females (n = 15), XY males (n = 15), and XXY males with Klinefelter syndrome (n = 35). The XY-iSEXS score in XXY-males was significantly lower than XX-females ($p < 2.2e-16$) but significantly higher than XY-males ($p = 0.0022$; Figure 3C). Importantly, the autosomal-iSEXS scores of XXY males were significantly higher than those of XY males ($p = 0.002$) but indistinguishable from those of XX females (Figure 3D). In GSE47584, a cohort of 10 males (5 XXY males with Klinefelter syndrome and 5 typical XY males), both XY-iSEXS and autosomal-iSEXS scores were significantly higher in XXY males than XY males (Figures S2A and S2B). Our comparison of XXY males and XY males in these two datasets identified 11 iSEXS genes significantly differentially expressed (FDR <5%; Figure S2C). Of those 11 genes, XXY males had increased expression of the five female-associated genes, three of which were known X-escape genes (*CA5BP1*, *CD40LG*, and *RPS4X*) (Figures S2D and S2E). XXY males also had decreased expression of the six male-associated genes, four of which play anti-microbial roles in neutrophil cytotoxic granules (*BPI*, *DEFA1B*, *DEFA4*, and *MPO*) (Figures S2F and S2G; Moraes et al., 2006; Schultz and Weiss, 2007). Thus, in two independent cohorts, we found that XXY males have consistently more “female-like” iSEXS gene expression, suggesting that the number of X chromosomes is associated with the autosomal-iSEXS score.

iSEXS Detects Higher Monocyte Proportions in Males

We investigated whether iSEXS genes were associated with specific immune cell types. For this purpose, for each gene in iSEXS, we used an immune-cell-type-specific effect size from the MetaSignature database (http://metasignature.stanford.edu) as described previously (Haynes et al., 2017; Vallania et al., 2018). Briefly, using 166 gene expression datasets composed of 6,160 human samples, representing 20 immune cell types, we estimated the specificity of expression of a gene in a particular immune cell type versus the other cell types as the Hedge’s *g*. We found that female-associated iSEXS genes were significantly enriched for genes highly expressed by $CD4^+$ T cells ($p = 0.0098$; Figure 4A). The male-associated iSEXS genes were significantly enriched for M0 macrophages ($p = 0.043$; Figure 4B). However, M0 macrophages are a rare cell type in the blood and thus are unlikely to drive transcriptomic sex differences. A more likely explanation is that male-associated iSEXS genes are highly expressed by myeloid cells in general, as evidenced by the two clusters of genes highly expressed by monocytes/macrophages and neutrophils/basophils (Figure 4B).

$CD4^+$ T cells and myeloid cells may be associated with iSEXS because of sex differences in immune cell proportions. Higher proportions of T cells in females have been reported previously, a possible explanation as to why female-associated iSEXS genes were enriched for genes expressed by $CD4^+$ cells (Melzer et al., 2015; Patin et al., 2018; Bouman et al., 2004; Carr et al., 2016; Lugada et al., 2004). To determine if a similar phenomenon

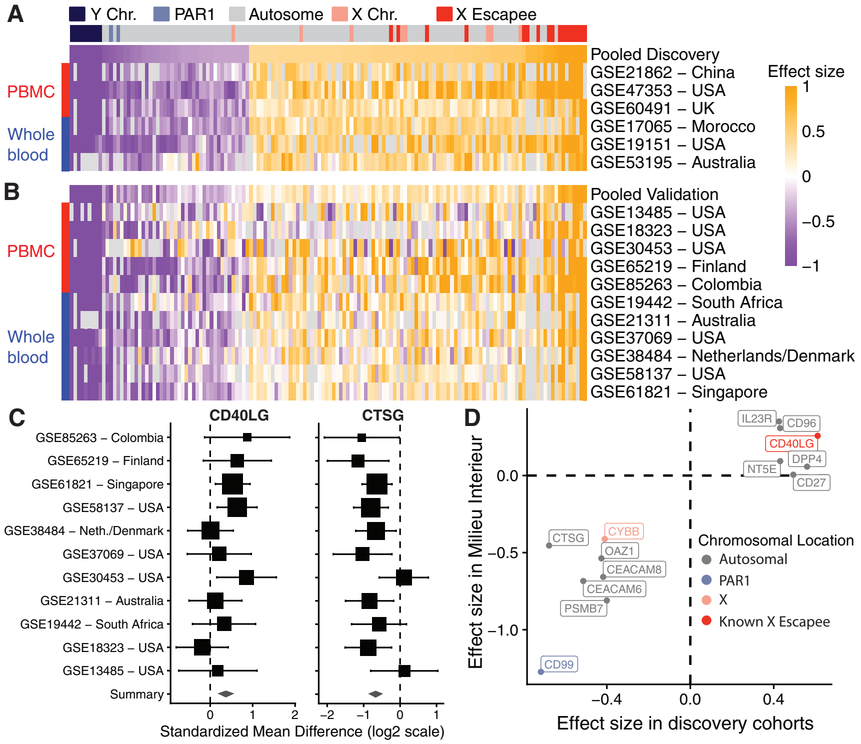

Figure 2. Effect Sizes of iSEXS in Discovery and Validation

(A and B) Heatmaps of effect sizes of iSEXS genes in (A) discovery and (B) validation cohorts. Each row is a dataset, and each column is a gene. The first row in each heatmap displays the pooled effect size across discovery or validation cohorts. Genes are ordered by increasing effect size in discovery cohorts in both heatmaps. The column color key indicates the chromosomal location of each gene (Y chromosome, dark blue; PAR1, light blue; autosome, gray; X chromosome, pink; known X-inactivation escape gene, red). The row color key indicates PBMC (red) or whole-blood (blue) datasets. Orange indicates a positive effect size for genes showing higher expression in females. Purple indicates a negative effect size for genes showing higher expression in males.

(C) Forest plots of *CD40LG* and *CTSG* effect sizes in the validation cohorts. PAR1 = pseudoautosomal region 1; PBMC = peripheral blood mononuclear cell; and Neth = Netherlands. The x axis represents standardized mean difference between females and males, computed as Hedge's g, in $\log_2$ scale. The size of aorti rectangle is inversely proportional to the standard error of mean in the corresponding study. Whiskers represent the 95% confidence interval. The diamond represents the overall, combined mean difference for a given gene. Width of the diamond represents the 95% confidence interval of overall mean difference.

(D) Comparison of the effect sizes of 13 iSEXS genes measured in the Milieu Interieur Consortium cohort of 279 healthy individuals 18-40 years old versus the effect sizes in discovery cohorts.

explained the association between male-associated iSEXS genes and myeloid cells, we investigated whether males had higher proportions of monocytes than females. To test this hypothesis, we used immune cell proportion data from the Milieu Intérieur Consortium cohort of 780 healthy individuals (391 females and 389 males) aged 20–70 years (Table 1). We found that among 18- to 40-year-old individuals, males had significantly higher monocyte percentages than females (p = 2.332e-11; Figure 4C).

Monocyte Proportions Increase in Older Females

Interestingly, in the Milieu Intérieur Consortium cohort, younger females had lower monocyte proportions than younger males, but older females had higher monocyte proportions that were statistically the same as those of older males (interaction between age and sex by two-way ANOVA p = 7.7e-04; Figure 4C). In contrast, monocyte proportions in younger males were the same as those in older males. We validated this observation in the independent Fragiadakis and Bjornson cohort (Fragiadakis et al., 2019; Table 1), profiled using cytometry by time of flight (CyTOF). In this cohort, younger males had significantly higher proportions of monocytes than younger females (p = 0.012; Figure S3A; Table 1), but there was no difference in monocyte proportions between older females and older males (Figure S3A; $p >$ 0.05). These results demonstrate that proportions of monocytes increase with age in females, but not males.

Higher proportions of monocytes in older females suggest that if male-associated autosomal-iSEXS genes are indeed preferentially expressed in monocytes, the autosomal-iSEXS score should also decrease in older female to the same level as older males. By combining three cohorts, we found that the autosomal-iSEXS scores in females decreased significantly after 50 years of age (p = 4.08e-5), but there was no significant difference between younger and older males (Figure 4D). In yet another independent cohort (ImmPort: SDY212), we found that the autosomal-iSEXS score was significantly higher in younger females than younger males (p = 4.5e-05), but there was no significant difference in the autosomal-iSEXS scores of older males and females (Figure S3B). Finally, in two additional cohorts (GSE65133 and GSE47353) of paired transcriptome and flow cytometry samples (Table 1), we observed statistically significant negative correlations between autosomal-iSEXS scores and monocyte proportions (GSE65133: $r = -0.79$, p = 0.2.9e-05; GSE47353: $r = -0.59$, p = 4.4e-06; Figures S3C and S3D). In contrast, the XY-iSEXS score did not correlate with monocyte proportions (Figures S3E and S3F) and did not change with age in females or males (Figure S3G).

Collectively, these results strongly suggest that (1) male-associated autosomal-iSEXS genes are preferentially expressed in monocytes, (2) younger females have lower monocyte percentages than younger males, and (3) percentages of monocytes increase with age in females to the same level as males. In addition, strong negative correlations between autosomal-iSEXS scores and monocyte percentages further suggest that iSEXS may be affected by differences in cellular proportions.

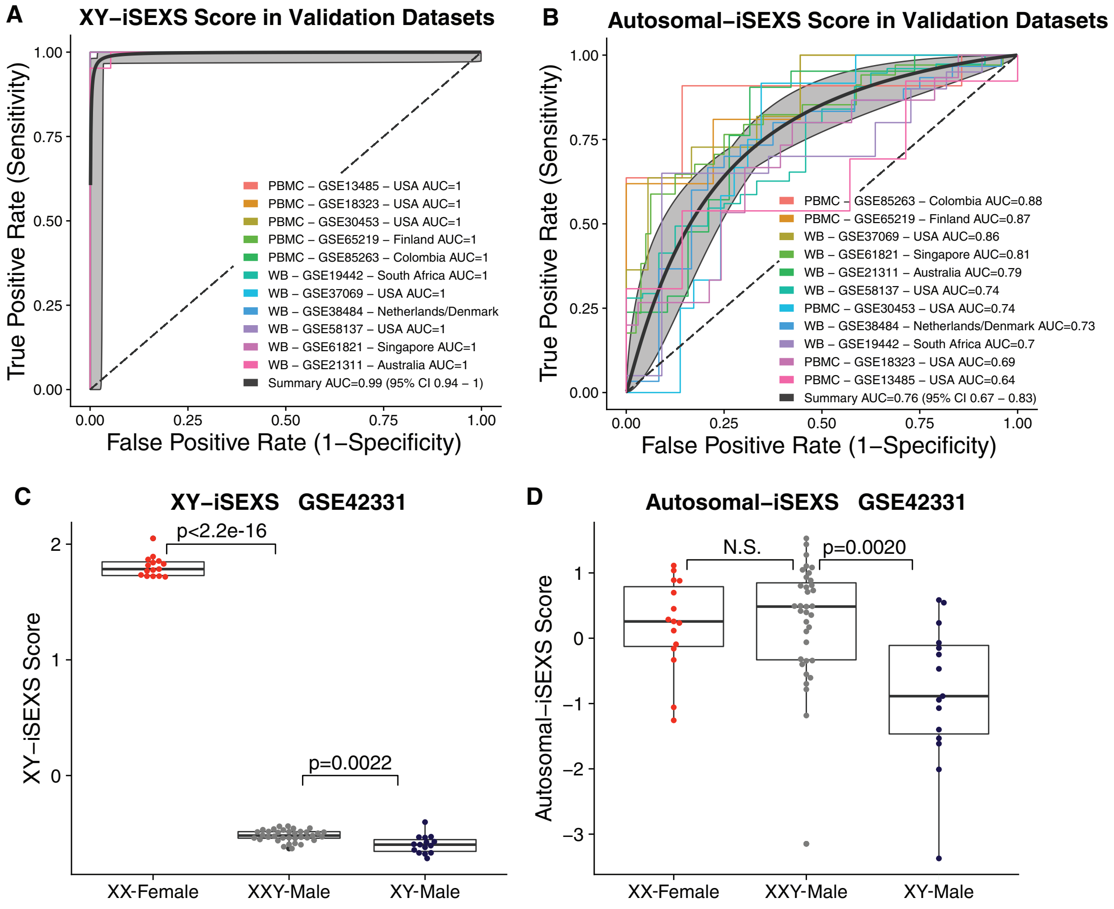

Figure 3. XY-iSEXS and Autosomal-iSEXS Performance in Typical Females, Typical Males, and Klinefelter Syndrome XXY Males

(A and B) ROC plots of performance of the (A) XY-iSEXS score (summary AUC 0.99 (95% CI 0.94-1.0)) and the (B) Autosomal-iSEXS score (summary AUC 0.76 (95% CI 0.67-0.83)) to differentiate males and females. Grey areas indicate 95% confidence intervals.

(C) Klinefelter syndrome XXY-males have significantly lower XY-iSEXS scores than XX females (t-test $p < 2.2e-16$) and significantly higher scores than XY-males (t-test $p = 0.0022$).

(D) There is no significant difference between Autosomal-iSEXS scores of XX-females and XXY-males, but XXY-males have significantly higher Autosomal-iSEXS scores than XY-males (t-test $p = 0.0020$).

See also Figures S1 and S2.

The Autosomal-iSEXS Score during Influenza Infection Reflects Changes in CD4$^+$ T Cell Proportions and Predicts Influenza Antibody Responses in Males

We utilized an influenza challenge study to explore how infection affects iSEXS. Liu et al. challenged healthy volunteers with H1N1 or H3N2 influenza virus and profiled their blood transcriptome for the subsequent 7 days (Liu et al., 2016). The autosomal-iSEXS score decreased within the first 50 h in males and females but rebounded in females earlier than males (Figure 5A), whereas XY-iSEXS scores remained unchanged in females and males following influenza infection (Figure S4). Next, we investigated whether CD4$^+$ T cells or monocytes were associated with changes in autosomal-iSEXS scores during influenza infection. As no cytometry data were available from influenza challenge studies, we estimated immune cell proportions from blood transcriptome data using cell mixture deconvolution. We used our previously developed basis matrix, immunoStates, to define immune cell subsets for deconvolution, as we have previously demonstrated its accuracy in health and disease (Vallania et al., 2018; Roy Chowdhury et al., 2018; Bongen et al., 2018; Scott et al., 2019). We found that, similar to autosomal-iSEXS scores, proportions of CD4$^+$ T cells decreased in both males and females following influenza infection but rebounded in females earlier than males (Figure 5B). In contrast, proportions of monocytes increased in both males and females following influenza infection until ~74 h and then returned to baseline levels (Figure 5C). This indicates that the autosomal-iSEXS score detects sex differences in the dynamics of the immune response to influenza infection, which may be explained by sex differences in CD4$^+$ T cell proportions.

In GSE68310, the whole-blood gene expression of volunteers was profiled at the beginning of flu season (baseline), during naturally acquired influenza A infection, and in the spring after flu season (Zhai et al., 2015). Antibody response was defined

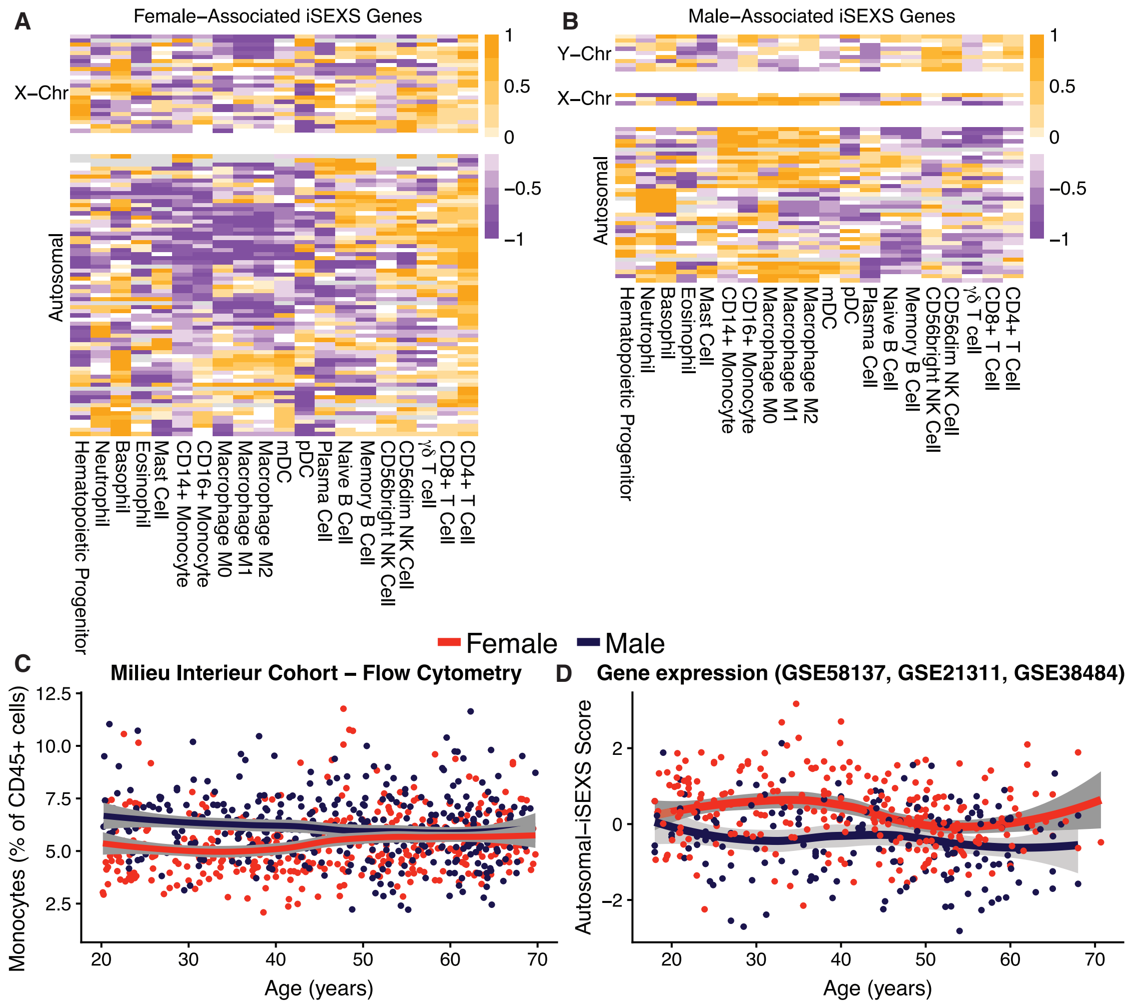

Figure 4. Cell-Type Enrichment of iSEXS Highlights CD4⁺ T Cells and Myeloid Cells

(A and B) Heatmaps of centered and scaled immune cell type specific effect sizes of (A) female-associated and (B) male-associated iSEXS genes. Orange indicates a high effect size, for cell types that highly express that gene. Purple indicates a low effect size, for cell types that express that gene less.

(C) Flow cytometry measured monocyte percentages of 816 healthy individuals from the Milieu Intérieur cohort. Two-way ANOVA was performed comparing the interaction of sex and age on monocyte proportions (sex p = 0.022; age p = 0.044; sex*age interaction p = 0.00077).

(D) Autosomal-iSEXS scores from 435 healthy individuals combined from GSE58137, GSE21311, and GSE38484. Two-way ANOVA was performed comparing the interaction of sex and age on autosomal-iSEXS scores (sex p = 0.00025; age p = 0.00013; sex*age interaction p = 0.18). t tests were performed comparing autosomal-iSEXS scores of younger (18–40 years old) versus older (≥50 years old) females (p = 4.08e-5) as well as younger versus older males (p = 0.15).

See also Figure S3.

as the difference in anti-H1N1 antibody titers between post-flu season and baseline (ΔTiters), with high and low responders defined as those with ΔTiters above or below the median, respectively. While the XY-iSEXS score at baseline did not correlate with ΔTiters in either females or males (Figure S5A), the autosomal-iSEXS score prior to infection (baseline) correlated with ΔTiters in males (p < 0.001, r = 0.74), but not in females (Figure 6A). Furthermore, the autosomal-iSEXS score at baseline predicted high- versus low-antibody-responder males (Figure 6B; AUC = 0.92, 95% CI 0.77-1.1) but not females (Figure 6B; AUC = 0.37, 95% CI 0.16-0.58). Notably, a strong correlation between the Autosomal-iSEXS scores before and after flu season was observed both in males (Figure 6C; r = 0.76, p = 0.00043) and females (Figure 6D; r = 0.66, p = 0.000044), suggesting that while the autosomal-iSEXS score changed during infection, some individuals may have consistently high or low autosomal-iSEXS scores in the healthy state. We also observed this consistency in the XY-iSEXS score, where pre- and post-influenza season scores were also significantly correlated in females and males (Figure S5B and S5C). We next asked whether the iSEXS scores were associated with antibody response to vaccination. Bucasas et al. (2011) profiled whole-blood transcriptional responses to influenza vaccination in males (GSE48018) and females (GSE48023) (Bucasas et al., 2011). We observed no significant correlations between either the XY-iSEXS score or autosomal-iSEXS score and vaccination antibody response (Figures S5D–S5G). We observed that the baseline autosomal-iSEXS score had a low correlation with vaccination antibody response

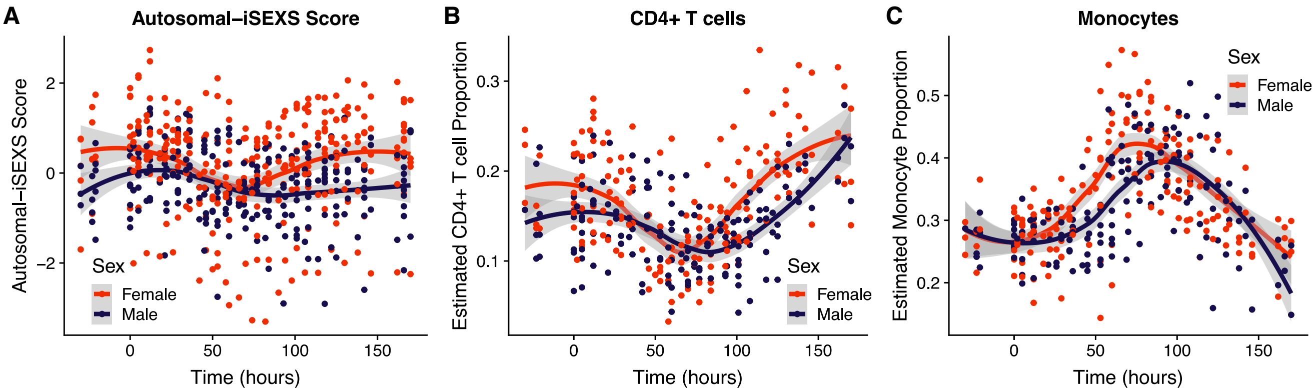

Figure 5. Differential Effect of Influenza Infection on Autosomal-iSEXS Score in Males and Females

In GSE73072, healthy volunteers were challenged with H1N1 or H3N2 influenza virus.

(A) Autosomal-iSEXS score is shown at the time of infection (hour 0) and the subsequent 7 days.

(B and C) Using immunoStates cell mixture deconvolution, (B) $CD4^+$ T cell and (C) monocyte proportions were estimated over the course of influenza infection. See Figure S4 for XY-iSEXS time course.

in males (Figure S5F; r = 0.18, p = 0.065) but no correlation in females (Figure S5G; r = −0.0063, p = 0.95). Taken together, these results suggest that the autosomal-iSEXS score captures biological factors relevant to antibody responses in males, but not females.

XY-iSEXS and Autosomal-iSEXS Scores Are Lower in Female SLE Patients

Finally, we investigated whether XY-iSEXS and autosomal-iSEXS also change in an autoimmune disease. We chose SLE, as it is a female-biased disease affecting nine females for every one male (Libert et al., 2010). In transcriptome data from two independent cohorts of patients with SLE, both the XY- and autosomal-iSEXS scores were significantly lower in female patients with SLE than female controls (Figures S6A and S6B). In contrast, there was no difference between the XY- and autosomal-iSEXS scores of male patients with SLE and male controls (Figures S6C and S6D). Both XY- and autosomal-iSEXS scores did not correlate with the SLE Disease Activity Index (SLEDAI) in either sex (Figures S6E and S6F). We next asked whether the iSEXS scores in SLE patients were associated with immune cell proportions. While monocyte proportions were not available, we observed that both XY- and autosomal-iSEXS scores were positively correlated with $CD4^+$ T cell percentages in female patients with SLE (XY-iSEXS: r = 0.6, p = 5.52e-5; autosomal-iSEXS: r = 0.35, p = 0.03; Figures S6G and S6H). In male patients with SLE, we observed no significant correlations with either the XY- or autosomal-iSEXS scores (Figures S6G and S6H). Overall, the iSEXS scores may change in SLE due to disease-related changes in $CD4^+$ T cell proportions.

DISCUSSION

We used whole-transcriptome profiles from genetically diverse healthy adults to identify a robust set of differentially expressed genes between males and females in white blood cells. Although several sex difference gene signatures have been reported, to the best of our knowledge, iSEXS is the first signature defined in multiple cohorts of different genetic and cultural backgrounds (Eady et al., 2005; Jansen et al., 2014; Melé et al., 2015; Piasecka et al., 2018; Whitney et al., 2003). Incorporating biological heterogeneity across datasets ensured that iSEXS (1) distinguishes males and females across diverse genetic backgrounds and (2) is not confounded by society-specific gender differences. Furthermore, we demonstrated that iSEXS is robust to technology as we validated iSEXS using gene expression data from RNA sequencing (RNA-seq), NanoString, and microarrays. Collectively, by defining iSEXS using diverse cohorts and validating it across biologically and technologically heterogeneous datasets, we ensured that iSEXS captured reproducible sex differences.

The 144 genes in iSEXS included genes with known immune function. Several lymphocyte-associated genes show higher expression in females (*CD40LG*, *LY9*, *CD27*, and *CD96*). Two members of the CEACAM family of cell adhesion molecules show higher expression in males (*CEACAM6* and *CEACAM8*) (Kuespert et al., 2006). Males had higher expression of seven genes that are known to be part of the phagocytic and extracellular anti-microbial responses of the innate immune system, including disrupting bacterial membrane integrity (*DEFA1*, *DEFA3*, *DEFA4*, and *BPI*), generating reactive oxygen species (*MPO*), and cleaving bacterial proteins (*ELANE* and *CTSG*) (Moraes et al., 2006). Furthermore, genes with increased expression in females were associated with adaptive responses, while genes with increased expression in males were associated with innate responses. The innate immune system may be protective against autoimmunity by clearing pathogens and dead cells that trigger self-reactions (Carroll, 2001). Whether this bias toward the adaptive or innate immune system between females and males exists beyond the transcriptome and whether it is relevant for susceptibility to autoimmune and infectious diseases require future laboratory-based experimentation.

Both biological and environmental factors can explain differences we observed between males and females in monocyte proportions and dynamics of $CD4^+$ T cells in response to influenza infection. Our analysis focused on integrating data from

OPEN ACCESS
CellPress

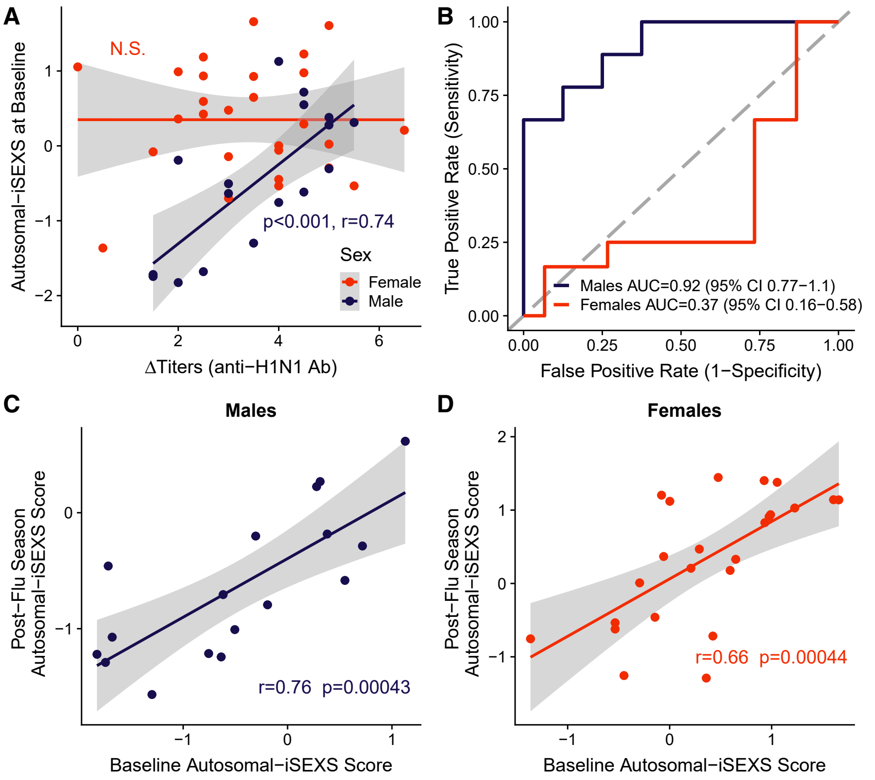

Figure 6. Autosomal-iSEXS Score Prior to Influenza Infection Predicts Antibody Response, but Only in Males

In GSE68310, healthy volunteers were followed at the beginning of flu season (baseline), during community-acquired influenza A infection and in the spring following flu season (post-flu season).

(A) Autosomal-iSEXS scores correlate with the change in anti-H1N1 antibody titers in males ($p < 0.001$, Pearson's $r = 0.74$), but not females. Grey area indicates a 95% confidence interval.

(B) Autosomal-iSEXS scores predict responder status in males (AUC = 0.92; 95% CI, 0.77-1.1), but not females (AUC = 0.37; 95% CI, 0.16-0.58).

(C and D) Male (C) and female (D) autosomal-iSEXS scores at baseline significantly correlate with post-flu season scores. Pearson's correlation coefficient and p value are given. Grey area indicates a 95% confidence interval.

See Figure S5 for XY-iSEXS score performance.

different countries to identify robust differences between males and females irrespective of ancestry and environmental factors. Hence, the differences in monocyte proportions and $CD4^+$ T cell dynamics due to ancestry or environmental factors are highly unlikely, although they cannot be completely ruled out due to lack of environmental data in our analysis.

In contrast, these consistent differences in monocytes and T cells between males and females are more likely due to a biological factor that is present universally across all datasets used in our analysis. A list of such factors includes (1) sex chromosomes and (2) hormones. Males with two X chromosomes (Klinefelter syndrome) are known to have increased estrogen and decreased testosterone levels. Hence, both factors are intrinsically linked to each other and impossible to conclusively deconvolve with available data. However, our analyses offer a possible explanation. First, our comparison of males with Klinefelter syndrome (XXY males) with XX females and XY males found that iSEXS is affected by the number of X chromosomes in an individual. The autosomal-iSEXS score was indistinguishable between XX females and XXY males. Importantly, the autosomal-iSEXS scores of XXY males were significantly higher than those of XY males ($p = 0.002$) but indistinguishable from those of XX females. Second, we found 11 genes in iSEXS (5 female associated and 6 male associated) were differentially expressed in XXY males compared to XY males in two independent datasets. Several of these genes, such as *BPI*, *CD40LG*, *MPO*, *DEFA1B*, and *DEFA4*, are known to be involved in immune responses. As XXY males have a female-like risk for SLE, our results suggest that the autosomal-iSEXS score may include aspects of the female immune system related to increased risk for SLE and other autoimmune diseases (Klein and Flanagan, 2016). Out of these 11 genes, 5 overexpressed genes in XXY males are expressed at higher levels in T cells than other immune cell subsets and are higher in XX females compared to XY males, whereas 5 underexpressed genes in XXY males are expressed at higher levels in monocytes and are higher in XY males than in XX females. Hence, these results suggest that differences in proportions of monocytes and $CD4^+$ T cell dynamics between females and males may be associated with the number of X chromosomes. However, the autosomal-iSEXS scores in older females were reduced to the same level as older males, although the number of X chromosomes remain the same in older women. This observation further suggests that the differences in proportions of monocytes and dynamics of $CD4^+$ T cells are more likely affected by hormones.

The transcriptional sex differences we observed may be partially explained by sex differences in proportions of $CD4^+$ T cells and monocytes (Piasecka et al., 2018). We found that female-associated iSEXS genes were enriched for $CD4^+$ T-cell-expressed genes, which may be because females have higher proportions of $CD4^+$ T cells in the blood (Bouman et al., 2004; Carr et al., 2016; Lugada et al., 2004; Melzer et al., 2015; Patin et al., 2018). We also observed that male-associated iSEXS genes were enriched for myeloid-cell-expressed genes, potentially due to age-dependent sex differences in monocyte proportions. We observed that younger females had lower monocyte proportions, but their monocyte proportions increased to male-like levels between 40 and 50 years of age. Our findings are supported by Boumen et al., who reported that younger females had lower monocyte counts than males, and Ben-Hur et al., who observed an increase in monocyte proportions with menopause in females that was reduced to premenopausal levels upon hormone replacement therapy (Ben-Hur et al., 1995; Bouman et al.,

2004). However, other studies did not observe sex differences in monocyte counts or proportions, possibly because menopause-associated aging changes were not fully investigated (Carr et al., 2016; Lugada et al., 2004; Patin et al., 2018; Seidler et al., 2010). This age-dependent change in monocyte proportions in older females, combined with effects of X dosage on autosomal-iSEXS, further suggests autosomal-iSEXS may be associated with and affected by changes in hormonal status in women. However, lack of hormone data in publicly available datasets used here does not allow such an analysis.

These sex differences in $CD4^+$ T cell and monocyte proportions are relevant for human health. We previously reported that baseline immune cell proportions may affect the outcome of influenza infection (Bongen et al., 2018). As both $CD4^+$ T cells and monocytes are important immune system regulators, differences at baseline may lead to differences during an immune response. Females have higher numbers of naive $CD4^+$ T cells and greater diversity of naive T cell receptor (TCR) repertoires, which may allow females to better develop memory against novel pathogens (Britanova et al., 2016). Both $CD4^+$ T cells and monocytes are important regulators of immune responses through cytokine production. Bouman et al. observed sex differences in cytokine production with higher intracellular levels of tumor necrosis factor (TNF), interleukin-1 (IL-1), and IL-12 in male monocytes and higher levels of IL-2 in female $CD3^+$ $CD8^-$ T cells (Bouman et al., 2004). The proportions of monocytes and $CD4^+$ T cells in the immune system at baseline may affect the outcome of an immune response by priming the immune system for a particular cytokine response. Furthermore, age-related changes in monocyte proportions may be relevant for autoimmunity. SLE is thought to be affected by menopause, because both disease activity and new-onset rates decrease after menopause (Khan and Ansar Ahmed, 2016; Sammaritano, 2012). Whether the changes in monocyte proportions with menopause play a role in SLE should be investigated in future studies.

Previous studies of influenza infection have highlighted stronger immune responses in females. In female mice, the median lethal dose of influenza is lower (Lorenzo et al., 2011), and immune responses to sublethal influenza infection are more severe (Vermillion et al., 2018). In humans, females generate stronger antibody responses and report more adverse events to influenza vaccination (Klein et al., 2015). We observed sex differences in the dynamics of $CD4^+$ T cell proportions during influenza infection. These $CD4^+$ T cell dynamics may be related to stronger immune responses and antibody titers in females, but future mechanistic studies are needed.

Interestingly, the autosomal-iSEXS score prior to influenza infection strongly correlated with antibody responses to influenza infection in males, but not females. We also observed a more modest correlation between influenza vaccination antibody responses in males, which may be because vaccination is a weaker immune stimulus than infection. It is unclear why the autosomal-iSEXS score did not correlate with influenza infection antibody responses in females. It is possible that the autosomal-iSEXS score captures some aspect of immune response that is relevant for antibody response in males, but not females (e.g., high testosterone levels) (Furman et al., 2014). Alternatively, the autosomal-iSEXS score may fluctuate with the menstrual cycle. If so, a female's menstrual cycle phase at the time of infection would be important, and thus the baseline autosomal-iSEXS score would have to be measured on the day of infection. Further studies specifically designed to examine the effect of hormone levels and menstrual cycle on iSEXS are needed.

While the autosomal-iSEXS score changed with influenza infection, in the healthy state, inter-individual variability was conserved. The individuals with the highest autosomal-iSEXS scores prior to flu season had the highest scores after flu season. This aligns with previous work demonstrating that immune cell proportions remain consistent in healthy individuals over the course of weeks to years (Brodin and Davis, 2017; Carr et al., 2016; Shen-Orr et al., 2016; Tsang et al., 2014). Our work indicates that the autosomal-iSEXS score is consistent over time in healthy individuals and may identify males who generate poor antibody responses to influenza infection. Thus, males with low autosomal-iSEXS scores in the healthy state may consistently produce poor antibody responses and may be at higher risk of influenza infection. Potentially, the autosomal-iSEXS score could be used to identify high-risk males who would benefit from extra precautions, such as higher influenza vaccination dosage or formulations that include an adjuvant.

We next investigated whether iSEXS is associated with autoimmunity using SLE. We observed that female patients with SLE had lower XY- and autosomal-iSEXS scores than female controls (Figure S6). This may be due to the fact that 15%–82% of SLE patients have lymphopenia, as reduced $CD4^+$ T cell proportions would result in reduced iSEXS scores (Carli et al., 2015). In fact, both the XY- and autosomal-iSEXS scores of female SLE patients correlated with $CD4^+$ T cell percentages (Figure S6). Larger cohorts are needed before we can draw conclusions about male patients with SLE. Overall, the iSEXS scores reflect differences in immune cell proportions between males and females as well as between SLE patients and healthy controls.

The bulk of our analyses focused on the autosomal-iSEXS score, but sex-chromosome genes also play critical roles in human health. Female-associated X-chromosome genes tend to be highly expressed in $CD4^+$ T cells, while male-associated X chromosome genes are highly expressed in myeloid cells (Figures 4A and 4B). Accordingly, we observed a negative correlation between the XY-iSEXS and monocyte percentages in one of two cohorts (Figure S5A and S5B). We observed no relationship between the XY-iSEXS score and $CD4^+$ T cell percentages, aging, influenza infection, or influenza titers (Figures S5C–S5G). This is expected considering that the XY-iSEXS score is dominated by the presence or absence of the Y chromosome, and thus nuanced effects of either sex chromosome are difficult to detect. We suggest that future studies examine these nuanced effects by separating XY-iSEXS genes by chromosomal location: Y chromosome, pseudoautosomal region, and X chromosome.

Our analyses have several limitations. First, we could not investigate the effect of menstrual cycle on iSEXS, because we only used publicly available data, and none of these studies recorded menstrual cycle phase in females. Second, the effect of sex hormones on iSEXS is also unclear, because no published studies have measured both hormone levels and blood transcriptome. Third, although we were able to examine iSEXS

patterns after menopause, we could not study pre-puberty iSEXS in children due to limited data.

We need to improve our understanding of the biological factors that underlie sex differences so that we do not rely on the crude labels of "male" and "female" when predicting disease risks. Our analysis demonstrates that iSEXS uses differences between the sexes to illuminate variation within the sexes. Not every female develops autoimmunity, and not every male succumbs to infection and cancer. Biological scores such as iSEXS that use sex differences to understand broader human variability could help predict disease risks in this age of precision medicine.

STAR★METHODS

Detailed methods are provided in the online version of this paper and include the following:

- KEY RESOURCES TABLE
- LEAD CONTACT AND MATERIALS AVAILABILITY
- METHOD DETAILS
  - Data Collection and Preprocessing
  - Dataset Selection for iSEXS Discovery and Validation Cohorts
  - Study Summaries
- QUANTIFICATION AND STATISTICAL ANALYSIS
  - Gene Expression Microarray Dataset Preprocessing
  - Imputing Sex Labels
  - Integrating Discovery Cohorts by Meta-Analysis
  - Statistical Power
  - Immune Sex Expression Signature and Score
  - Chromosome Localization
  - Immune Cell Specificity Scores and Cellular Deconvolution
  - Statistical Tests
- DATA AND CODE AVAILABILITY

SUPPLEMENTAL INFORMATION

Supplemental Information can be found online at https://doi.org/10.1016/j.celrep.2019.10.019.

ACKNOWLEDGMENTS

We thank Greg Gibson and Monica Rojas-Pena for assistance with GSE21311 and GSE85263 and Francesco Vallania, Marcia Stefanick, and Kelly McGill for helpful conversations. This work was supported in part by the Autoimmunity Center of Excellence (grants 5U19AI110491-04 and 5UM1A110498-04), a research consortium supported by the NIAID/NIH (P.J.U.), the Bill & Melinda Gates Foundation (P.K.), the Donald E. and Delia B. Baxter Foundation (P.J.U.), Elizabeth F. Adler (P.J.U.), the Henry Gustav Floren Trust (P.J.U.), the NIH (grants 5U19AI109662-05 and 5U19AI057229 to P.K. and grant 5R01AI125197-02 to P.J.U. and P.K.), the Department of Defense (contracts W81XWH-18-1-0253 and W81XWH1910235 to P.K.), the Ralph & Marian Falk Medical Research Trust (P.K.), and NIH Clinical and Translational Science Award UL1 RR025744 (Stanford Center for Clinical and Translational Education and Research; Spectrum). E.B. was supported by the Stanford Gabilan Graduate Fellowship in Science and Engineering and a Stanford Women and Sex Differences in Medicine (WSDM) seed grant. G.K.F., Z.B.B., and G.P.N. were supported in part by the U.S. Food and Drug Administration (contract HHSF223201210194C). G.K.F. was supported by the Stanford Bio-X graduate research fellowship (Stanford, CA, USA) and the NIH (T32GM007276; Bethesda, MD, USA). This article reflects the views of the authors and should not be construed to represent the U.S. Food and Drug Administration's views or policies.

AUTHOR CONTRIBUTIONS

E.B. conceived the study. E.B., P.J.U., and P.K. designed experiments. E.B. performed the data analysis under the supervision of P.J.U. and P.K. H.L. and A.K. identified aging cohorts and assisted with analysis. G.K.F., Z.B.B., and G.P.N. generated Fragiadakis and Bjornson cohort data. E.B., P.J.U., and P.K. jointly interpreted the results and wrote the manuscript with contributions from all co-authors. All authors read and approved the final manuscript.

DECLARATION OF INTERESTS

The authors declare no competing interests.

Received: March 27, 2019
Revised: July 12, 2019
Accepted: October 3, 2019
Published: November 12, 2019

REFERENCES

Andres-Terre, M., McGuire, H.M., Pouliot, Y., Bongen, E., Sweeney, T.E., Tato, C.M., and Khatri, P. (2015). Integrated, multi-cohort analysis identifies conserved transcriptional signatures across multiple respiratory viruses. Immunity *43*, 1199–1211.

Barrett, T., Wilhite, S.E., Ledoux, P., Evangelista, C., Kim, I.F., Tomashevsky, M., Marshall, K.A., Phillippy, K.H., Sherman, P.M., Holko, M., et al. (2013). NCBI GEO: archive for functional genomics data sets–update. Nucleic Acids Res. *41*, D991–D995.

Ben-Hur, H., Mor, G., Insler, V., Blickstein, I., Amir-Zaltsman, Y., Sharp, A., Globerson, A., and Kohen, F. (1995). Menopause is associated with a significant increase in blood monocyte number and a relative decrease in the expression of estrogen receptors in human peripheral monocytes. Am. J. Reprod. Immunol. *34*, 363–369.

Benjamini, Y., and Hochberg, Y. (1995). Controlling the false discovery rate: a practical and powerful approach to multiple testing. J. R. Stat. Soc. B Stat. Methodol. *57*, 289–300.

Berry, M.P., Graham, C.M., McNab, F.W., Xu, Z., Bloch, S.A., Oni, T., Wilkinson, K.A., Banchereau, R., Skinner, J., Wilkinson, R.J., et al. (2010). An interferon-inducible neutrophil-driven blood transcriptional signature in human tuberculosis. Nature *466*, 973–977.

Bhattacharya, S., Dunn, P., Thomas, C.G., Smith, B., Schaefer, H., Chen, J., Hu, Z., Zalocusky, K.A., Shankar, R.D., Shen-Orr, S.S., et al. (2018). ImmPort, toward repurposing of open access immunological assay data for translational and clinical research. Sci. Data *5*, 180015.

Bongen, E., Vallania, F., Utz, P.J., and Khatri, P. (2018). KLRD1-expressing natural killer cells predict influenza susceptibility. Genome Med. *10*, 45.

Bouman, A., Schipper, M., Heineman, M.J., and Faas, M.M. (2004). Gender difference in the non-specific and specific immune response in humans. Am. J. Reprod. Immunol. *52*, 19–26.

Britanova, O.V., Shugay, M., Merzlyak, E.M., Staroverov, D.B., Putintseva, E.V., Turchaninova, M.A., Mamedov, I.Z., Pogorelyy, M.V., Bolotin, D.A., Izraelson, M., et al. (2016). Dynamics of individual T cell repertoires: from cord blood to centenarians. J. Immunol. *196*, 5005–5013.

Brodin, P., and Davis, M.M. (2017). Human immune system variation. Nat. Rev. Immunol. *17*, 21–29.

Bucasas, K.L., Franco, L.M., Shaw, C.A., Bray, M.S., Wells, J.M., Niño, D., Arden, N., Quarles, J.M., Couch, R.B., and Belmont, J.W. (2011). Early patterns of gene expression correlate with the humoral immune response to influenza vaccination in humans. J. Infect. Dis. *203*, 921–929.

Carli, L., Tani, C., Vagnani, S., Signorini, V., and Mosca, M. (2015). Leukopenia, lymphopenia, and neutropenia in systemic lupus erythematosus: prevalence and clinical impact: a systematic literature review. Semin. Arthritis Rheum. *45*, 190–194.

Carlson, Marc (2016). org.Hs.eg.db: Genome wide annotation for Human. R package version 3.4.0.

Carr, E.J., Dooley, J., Garcia-Perez, J.E., Lagou, V., Lee, J.C., Wouters, C., Meyts, I., Goris, A., Boeckxstaens, G., Linterman, M.A., and Liston, A. (2016). The cellular composition of the human immune system is shaped by age and cohabitation. Nat. Immunol. *17*, 461–468.

Carroll, M. (2001). Innate immunity in the etiopathology of autoimmunity. Nat. Immunol. *2*, 1089–1090.

Chiche, L., Jourde-Chiche, N., Whalen, E., Presnell, S., Gersuk, V., Dang, K., Anguiano, E., Quinn, C., Burtey, S., Berland, Y., Kaplanski, G., Harle, J.R., Pascual, V., and Chaussabel, D. (2014). Modular transcriptional repertoire analyses of adults with systemic lupus erythematosus reveal distinct type I and type II interferon signatures. Arthritis Rheumatol. *66*, 1583–1595.

Cook, M.B., McGlynn, K.A., Devesa, S.S., Freedman, N.D., and Anderson, W.F. (2011). Sex disparities in cancer mortality and survival. Cancer Epidemiol. Biomarkers Prev. *20*, 1629–1637.

de Jong, S., Boks, M.P., Fuller, T.F., Strengman, E., Janson, E., de Kovel, C.G., Ori, A.P., Vi, N., Mulder, F., Blom, J.D., et al. (2012). A gene co-expression network in whole blood of schizophrenia patients is independent of antipsychotic-use and enriched for brain-expressed genes. PLoS ONE *7*, e39498.

Dorak, M.T., and Karpuzoglu, E. (2012). Gender differences in cancer susceptibility: an inadequately addressed issue. Front. Genet. *3*, 268.

Durinck, S., Spellman, P.T., Birney, E., and Huber, W. (2009). Mapping identifiers for the integration of genomic datasets with the R/Bioconductor package biomaRt. Nat. Protoc. *4*, 1184–1191.

Eady, J.J., Wortley, G.M., Wormstone, Y.M., Hughes, J.C., Astley, S.B., Foxall, R.J., Doleman, J.F., and Elliott, R.M. (2005). Variation in gene expression profiles of peripheral blood mononuclear cells from healthy volunteers. Physiol. Genomics *22*, 402–411.

Fisher, R.A. (1934). Statistical Methods for Research Workers (Oliver & Boyd).

Fragiadakis, G.K., Bjornson, Z.B., Madhireddy, D., Sachs, K., Spitzer, M.H., Bendall, S.C., and Nolan, G.P. (2019). Variation of immune cell responses in humans reveals sex-specific coordinated signaling across cell types. bioRxiv. https://doi.org/10.1101/567784.

Furman, D., Jojic, V., Kidd, B., Shen-Orr, S., Price, J., Jarrell, J., Tse, T., Huang, H., Lund, P., Maecker, H.T., et al. (2013). Apoptosis and other immune biomarkers predict influenza vaccine responsiveness. Mol. Syst. Biol. *9*, 659.

Furman, D., Hejblum, B.P., Simon, N., Jojic, V., Dekker, C.L., Thiébaut, R., Tibshirani, R.J., and Davis, M.M. (2014). Systems analysis of sex differences reveals an immunosuppressive role for testosterone in the response to influenza vaccination. Proc. Natl. Acad. Sci. USA *111*, 869–874.

Groth, K.A., Skakkebæk, A., Høst, C., Gravholt, C.H., and Bojesen, A. (2013). Clinical review: Klinefelter syndrome—a clinical update. J. Clin. Endocrinol. Metab. *98*, 20–30.

Haynes, W.A., Vallania, F., Liu, C., Bongen, E., Tomczak, A., Andres-Terrè, M., Lofgren, S., Tam, A., Deisseroth, C.A., Li, M.D., et al. (2017). Empowering multi-cohort gene expression analysis to increase reproducibility. Pac. Symp. Biocomput. *22*, 144–153.

Hedges, L.V., and Pigott, T.D. (2001). The power of statistical tests in meta-analysis. Psychol. Methods *6*, 203–217.

Heinzen, E.L., Ge, D., Cronin, K.D., Maia, J.M., Shianna, K.V., Gabriel, W.N., Welsh-Bohmer, K.A., Hulette, C.M., Denny, T.N., and Goldstein, D.B. (2008). Tissue-specific genetic control of splicing: implications for the study of complex traits. PLoS Biol. *6*, e1.

Hemani, G., Shakhbazov, K., Westra, H.J., Esko, T., Henders, A.K., McRae, A.F., Yang, J., Gibson, G., Martin, N.G., Metspalu, A., et al. (2014). Detection and replication of epistasis influencing transcription in humans. Nature *508*, 249–253.

Hoang, L.T., Tolfvenstam, T., Ooi, E.E., Khor, C.C., Naim, A.N., Ho, E.X., Ong, S.H., Wertheim, H.F., Fox, A., Van Vinh Nguyen, C., et al. (2014). Patient-based transcriptome-wide analysis identify interferon and ubiquination pathways as potential predictors of influenza A disease severity. PLoS ONE *9*, e111640.

Huang, J., Zhang, L., Deng, H., Chang, L., Liu, Q., and Liu, P. (2015). Global transcriptome analysis of peripheral blood identifies the most significantly down-regulated genes associated with metabolism regulation in Klinefelter syndrome. Mol. Reprod. Dev. *82*, 17–25.

Idaghdour, Y., Czika, W., Shianna, K.V., Lee, S.H., Visscher, P.M., Martin, H.C., Miclaus, K., Jadallah, S.J., Goldstein, D.B., Wolfinger, R.D., and Gibson, G. (2010). Geographical genomics of human leukocyte gene expression variation in southern Morocco. Nat. Genet. *42*, 62–67.

Jansen, R., Batista, S., Brooks, A.I., Tischfield, J.A., Willemsen, G., van Grootheest, G., Hottenga, J.J., Milaneschi, Y., Mbarek, H., Madar, V., et al. (2014). Sex differences in the human peripheral blood transcriptome. BMC Genomics *15*, 33.

Khan, D., and Ansar Ahmed, S. (2016). The immune system is a natural target for estrogen action: opposing effects of estrogen in two prototypical autoimmune diseases. Front. Immunol. *6*, 635.

Khatri, P., Roedder, S., Kimura, N., De Vusser, K., Morgan, A.A., Gong, Y., Fischbein, M.P., Robbins, R.C., Naesens, M., Butte, A.J., and Sarwal, M.M. (2013). A common rejection module (CRM) for acute rejection across multiple organs identifies novel therapeutics for organ transplantation. J. Exp. Med. *210*, 2205–2221.

Klein, S.L., and Flanagan, K.L. (2016). Sex differences in immune responses. Nat. Rev. Immunol. *16*, 626–638.

Klein, S.L., Marriott, I., and Fish, E.N. (2015). Sex-based differences in immune function and responses to vaccination. Trans. R. Soc. Trop. Med. Hyg. *109*, 9–15.

Kuespert, K., Pils, S., and Hauck, C.R. (2006). CEACAMs: their role in physiology and pathophysiology. Curr. Opin. Cell Biol. *18*, 565–571.

Kuparinen, T., Marttila, S., Jylhävä, J., Tserel, L., Peterson, P., Jylhä, M., Hervonen, A., and Hurme, M. (2013). Cytomegalovirus (CMV)-dependent and -independent changes in the aging of the human immune system: a transcriptomic analysis. Exp. Gerontol. *48*, 305–312.

Lauwerys, B.R., Hachullam, E., Spertini, F., Lazaro, E., Jorgensen, C., Mariette, X., Haelterman, E., Grouard-Vogel, G., Fanget, B., Dhellin, O., Vandepapelière, P., and Houssiau, F.A. (2013). Down-regulation of interferon signature in systemic lupus erythematosus patients by active immunization with interferon α-kinoid. Arthritis Rheum. *65*, 447–456.

Lewis, D.A., Stashenko, G.J., Akay, O.M., Price, L.I., Owzar, K., Ginsburg, G.S., Chi, J.T., and Ortel, T.L. (2011). Whole blood gene expression analyses in patients with single versus recurrent venous thromboembolism. Thromb. Res. *128*, 536–540.

Liang, Y., Tsoi, L.C., Xing, X., Beamer, M.A., Swindell, W.R., Sarkar, M.K., Berthier, C.C., Stuart, P.E., Harms, P.W., Nair, R.P., et al. (2017). A gene network regulated by the transcription factor VGLL3 as a promoter of sex-biased autoimmune diseases. Nat. Immunol. *18*, 152–160.

Libert, C., Dejager, L., and Pinheiro, I. (2010). The X chromosome in immune functions: when a chromosome makes the difference. Nat. Rev. Immunol. *10*, 594–604.

Liu, T.Y., Burke, T., Park, L.P., Woods, C.W., Zaas, A.K., Ginsburg, G.S., and Hero, A.O. (2016). An individualized predictor of health and disease using paired reference and target samples. BMC Bioinformatics *17*, 47.

Lorenzo, M.E., Hodgson, A., Robinson, D.P., Kaplan, J.B., Pekosz, A., and Klein, S.L. (2011). Antibody responses and cross protection against lethal influenza A viruses differ between the sexes in C57BL/6 mice. Vaccine *29*, 9246–9255.

Lugada, E.S., Mermin, J., Kaharuza, F., Ulvestad, E., Were, W., Langeland, N., Asjo, B., Malamba, S., and Downing, R. (2004). Population-based hematologic and immunologic reference values for a healthy Ugandan population. Clin. Diagn. Lab. Immunol. *11*, 29–34.

Mason, E., Tronc, G., Nones, K., Matigian, N., Kim, J., Aronow, B.J., Wolfinger, R.D., Wells, C., and Gibson, G. (2010). Maternal influences on the transmission of leukocyte gene expression profiles in population samples from Brisbane, Australia. PLoS ONE *5*, e14479.

McHale, C.M., Zhang, L., Lan, Q., Vermeulen, R., Li, G., Hubbard, A.E., Porter, K.E., Thomas, R., Portier, C.J., Shen, M., et al. (2011). Global gene expression profiling of a population exposed to a range of benzene levels. Environ. Health Perspect. *119*, 628–634.

Melé, M., Ferreira, P.G., Reverter, F., DeLuca, D.S., Monlong, J., Sammeth, M., Young, T.R., Goldmann, J.M., Pervouchine, D.D., Sullivan, T.J., et al.; GTEx Consortium (2015). Human genomics. The human transcriptome across tissues and individuals. Science *348*, 660–665.

Melzer, S., Zachariae, S., Bocsi, J., Engel, C., Löffler, M., and Tárnok, A. (2015). Reference intervals for leukocyte subsets in adults: Results from a population-based study using 10-color flow cytometry. Cytometry B Clin. Cytom. *88*, 270–281.

Moraes, T.J., Zurawska, J.H., and Downey, G.P. (2006). Neutrophil granule contents in the pathogenesis of lung injury. Curr. Opin. Hematol. *13*, 21–27.

Newman, A.M., Liu, C.L., Green, M.R., Gentles, A.J., Feng, W., Xu, Y., Hoang, C.D., Diehn, M., and Alizadeh, A.A. (2015). Robust enumeration of cell subsets from tissue expression profiles. Nat. Methods *12*, 453–457.

Owens, I.P. (2002). Ecology and evolution. Sex differences in mortality rate. Science *297*, 2008–2009.

Patin, E., Hasan, M., Bergstedt, J., Rouilly, V., Libri, V., Urrutia, A., Alanio, C., Scepanovic, P., Hammer, C., Jönsson, F., et al.; Milieu Intérieur Consortium (2018). Natural variation in the parameters of innate immune cells is preferentially driven by genetic factors. Nat. Immunol. *19*, 302–314.

Pennell, L.M., Galligan, C.L., and Fish, E.N. (2012). Sex affects immunity. J. Autoimmun *38*, J282–J291.

Peters, M.J., Joehanes, R., Pilling, L.C., Schurmann, C., Conneely, K.N., Powell, J., Reinmaa, E., Sutphin, G.L., Zhernakova, A., Schramm, K., et al. (2015). The transcriptional landscape of age in human peripheral blood. Nat. Commun. *6*, 8570.

Piasecka, B., Duffy, D., Urrutia, A., Quach, H., Patin, E., Posseme, C., Bergstedt, J., Charbit, B., Rouilly, V., MacPherson, C.R., et al.; Milieu Intérieur Consortium (2018). Distinctive roles of age, sex, and genetics in shaping transcriptional variation of human immune responses to microbial challenges. Proc. Natl. Acad. Sci. USA *115*, E488–E497.

Querec, T.D., Akondy, R.S., Lee, E.K., Cao, W., Nakaya, H.I., Teuwen, D., Pirani, A., Gernert, K., Deng, J., Marzolf, B., et al. (2009). Systems biology approach predicts immunogenicity of the yellow fever vaccine in humans. Nat. Immunol. *10*, 116–125.

Ramasamy, A., Mondry, A., Holmes, C.C., and Altman, D.G. (2008). Key issues in conducting a meta-analysis of gene expression microarray datasets. PLoS Med. *5*, e184.

Rojas-Pena, M.C., Arafat, D.C., Velasquez, J.M., Garimalla, S.C., Arevalo-Herrera, M., Herrera, S., and Gibson, G. (2018). Profiling gene expression of the host response to a Plasmodium vivax irradiated sporozoite immunization and infectious challenge. bioRxiv. https://doi.org/10.1101/501957.

Roy Chowdhury, R., Vallania, F., Yang, Q., Lopez Angel, C.J., Darboe, F., Penn-Nicholson, A., Rozot, V., Nemes, E., Malherbe, S.T., Ronacher, K., et al. (2018). A multi-cohort study of the immune factors associated with M. tuberculosis infection outcomes. Nature *560*, 644–648.

Sammaritano, L.R. (2012). Menopause in patients with autoimmune diseases. Autoimmun. Rev. *11*, A430–A436.

Sasidhar, M.V., Itoh, N., Gold, S.M., Lawson, G.W., and Voskuhl, R.R. (2012). The XX sex chromosome complement in mice is associated with increased spontaneous lupus compared with XY. Ann. Rheum. Dis. *71*, 1418–1422.

Schultz, H., and Weiss, J.P. (2007). The bactericidal/permeability-increasing protein (BPI) in infection and inflammatory disease. Clin. Chim. Acta *384*, 12–23.

Scott, M.K.D., Quinn, K., Li, Q., Carroll, R., Warsinske, H., Vallania, F., Chen, S., Carns, M.A., Aren, K., Sun, J., et al. (2019). Increased monocyte count as a cellular biomarker for poor outcomes in fibrotic diseases: a retrospective, multicentre cohort study. Lancet Respir. Med. *7*, 497–508.

Seidler, S., Zimmermann, H.W., Bartneck, M., Trautwein, C., and Tacke, F. (2010). Age-dependent alterations of monocyte subsets and monocyte-related chemokine pathways in healthy adults. BMC Immunol. *11*, 30.

Seok, J., Warren, H.S., Cuenca, A.G., Mindrinos, M.N., Baker, H.V., Xu, W., Richards, D.R., McDonald-Smith, G.P., Gao, H., Hennessy, L., et al.; Inflammation and Host Response to Injury, Large Scale Collaborative Research Program (2013). Genomic responses in mouse models poorly mimic human inflammatory diseases. Proc. Natl. Acad. Sci. USA *110*, 3507–3512.

Shen-Orr, S.S., Furman, D., Kidd, B.A., Hadad, F., Lovelace, P., Huang, Y.W., Rosenberg-Hasson, Y., Mackey, S., Grisar, F.A., Pickman, Y., et al. (2016). Defective signaling in the JAK-STAT pathway tracks with chronic inflammation and cardiovascular risk in aging humans. Cell Syst. *3*, 374–384.e4.

Souyris, M., Cenac, C., Azar, P., Daviaud, D., Canivet, A., Grunenwald, S., Pienkowski, C., Chaumeil, J., Mejía, J.E., and Guéry, J.C. (2018). *TLR7* escapes X chromosome inactivation in immune cells. Sci. Immunol. *3*, 3.

Toker, L., Feng, M., and Pavlidis, P. (2016). Whose sample is it anyway? Widespread misannotation of samples in transcriptomics studies. F1000Res. *5*, 2103.

Tsang, J.S., Schwartzberg, P.L., Kotliarov, Y., Biancotto, A., Xie, Z., Germain, R.N., Wang, E., Olnes, M.J., Narayanan, M., Golding, H., et al.; Baylor HIPC Center; CHI Consortium (2014). Global analyses of human immune variation reveal baseline predictors of postvaccination responses. Cell *157*, 499–513.

Tukiainen, T., Villani, A.C., Yen, A., Rivas, M.A., Marshall, J.L., Satija, R., Aguirre, M., Gauthier, L., Fleharty, M., Kirby, A., et al.; GTEx Consortium; Laboratory, Data Analysis &Coordinating Center (LDACC)—Analysis Working Group; Statistical Methods groups—Analysis Working Group; Enhancing GTEx (eGTEx) groups; NIH Common Fund; NIH/NCI; NIH/NHGRI; NIH/NIMH; NIH/NIDA; Biospecimen Collection Source Site—NDRI; Biospecimen Collection Source Site—RPCI; Biospecimen Core Resource—VARI; Brain Bank Repository—University of Miami Brain Endowment Bank; Leidos Biomedical—Project Management; ELSI Study; Genome Browser Data Integration &Visualization—EBI; Genome Browser Data Integration &Visualization—UCSC Genomics Institute, University of California Santa Cruz (2017). Landscape of X chromosome inactivation across human tissues. Nature *550*, 244–248.

Vahey, M.T., Wang, Z., Kester, K.E., Cummings, J., Heppner, D.G., Jr., Nau, M.E., Ofori-Anyinam, O., Cohen, J., Coche, T., Ballou, W.R., and Ockenhouse, C.F. (2010). Expression of genes associated with immunoproteasome processing of major histocompatibility complex peptides is indicative of protection with adjuvanted RTS,S malaria vaccine. J. Infect. Dis. *201*, 580–589.

Vallania, F., Tam, A., Lofgren, S., Schaffert, S., Azad, T.D., Bongen, E., Haynes, W., Alsup, M., Alonso, M., Davis, M., et al. (2018). Leveraging heterogeneity across multiple datasets increases cell-mixture deconvolution accuracy and reduces biological and technical biases. Nat. Commun. *9*, 4735.

Vedhara, K., Gill, S., Eldesouky, L., Campbell, B.K., Arevalo, J.M., Ma, J., and Cole, S.W. (2015). Personality and gene expression: Do individual differences exist in the leukocyte transcriptome? Psychoneuroendocrinology *52*, 72–82.

Vermillion, M.S., Ursin, R.L., Kuok, D.I.T., Vom Steeg, L.G., Wohlgemuth, N., Hall, O.J., Fink, A.L., Sasse, E., Nelson, A., Ndeh, R., et al. (2018). Production of amphiregulin and recovery from influenza is greater in males than females. Biol. Sex Differ. *9*, 24.

Whitney, A.R., Diehn, M., Popper, S.J., Alizadeh, A.A., Boldrick, J.C., Relman, D.A., and Brown, P.O. (2003). Individuality and variation in gene expression patterns in human blood. Proc. Natl. Acad. Sci. USA *100*, 1896–1901.

Zhai, Y., Franco, L.M., Atmar, R.L., Quarles, J.M., Arden, N., Bucasas, K.L., Wells, J.M., Niño, D., Wang, X., Zapata, G.E., et al. (2015). Host transcriptional response to influenza and other acute respiratory viral infections: a prospective cohort study. PLoS Pathog. *11*, e1004869.

Zitzmann, M., Bongers, R., Werler, S., Bogdanova, N., Wistuba, J., Kliesch, S., Gromoll, J., and Tüttelmann, F. (2015). Gene expression patterns in relation to the clinical phenotype in Klinefelter syndrome. J. Clin. Endocrinol. Metab. *100*, E518–E523.

STAR★METHODS

KEY RESOURCES TABLE

REAGENT or RESOURCE	SOURCE	IDENTIFIER
Software and Algorithms		
MetaIntegrator	R package	https://cran.r-project.org/web/packages/MetaIntegrator/index.html
Deconvolution Analysis (within MetaIntegrator)	R package	Vallania et al. (2018)
biomaRt	R package	https://bioconductor.org/packages/release/bioc/html/biomaRt.html

LEAD CONTACT AND MATERIALS AVAILABILITY

While this study generated no biological reagents, requests concerning methods and computational resources will be fulfilled by the Lead Contact, Dr. Purvesh Khatri (pkhatri@stanford.edu).

METHOD DETAILS

Data Collection and Preprocessing

We downloaded 27 whole transcriptome datasets using microarray, RNA-seq, or Nanostring from NCBI Gene Expression Omnibus (GEO), ImmPort (Barrett et al., 2013; Bhattacharya et al., 2018), and the Milieu Interieur Consortium (Barrett et al., 2013; Bhattacharya et al., 2018; Piasecka et al., 2018). These datasets consisted of 2,639 whole blood (WB) or Peripheral Blood Mononuclear Cells (PBMC) samples from human adults. Of these 2,639 samples, 971 samples were from 18-to-40-year old healthy individuals, defined as those without cancer, autoimmune disease, or active infection. We supplemented our analysis with 943 samples profiled using cytometry in four independent cohorts (Table 1). These datasets encompassed broad technical heterogeneity represented by 13 microarray platforms from 3 manufacturers, and use of both flow and mass cytometry. Importantly, these datasets encompassed broad biological heterogeneity by using datasets represented by six continents and 17 countries (Australia, Belgium, Bulgaria, China, Colombia, Croatia, Denmark, Finland, France, Germany, Morocco, Netherlands, Singapore, South Africa, Switzerland, UK, and USA).

Dataset Selection for iSEXS Discovery and Validation Cohorts

The Discovery and Validation cohorts contained datasets from human whole blood (WB) or Peripheral Blood Mononuclear cells (PBMCs), from healthy adults aged between 18 and 40 years old. Using the R package, GEOquery (citation), we identified human gene expression microarray and RNaseq datasets in WB or PBMCs. We excluded disease samples from both Discovery and Validation cohorts. If sex or age labels were not present, we obtained them in correspondence with the original authors. If sex was identified, but not age, then we included the dataset if all samples were within the age range as reported by the original publication. Discovery datasets were chosen as those with >40 samples, known sex and age labels, and worldwide representation of human populations. Sex annotations and age ranges were available for all discovery datasets. We were unable to obtain sex or age annotations for a few validation datasets (Study Summaries).

Study Summaries

Discovery Cohorts

GSE17065: Idaghdour et al. (2010) collected peripheral blood samples from Arab and Amazigh Berber populations from both rural and urban areas of Morocco. They profiled gene expression in 208 individuals, ranging in age from 17 to 69 years old. We removed 13 individuals due to conflict between the author-supplied sex annotation and transcriptionally imputed sex labels.

GSE19151: Lewis et al. (2011) examined whole blood gene expression samples from 70 adults with venous thromboembolism on warfarin and 63 healthy controls. We excluded individuals with venous thromboembolism from our analysis. We used healthy controls 18-40 years old as a discovery cohort. We excluded three individuals due to conflicting sex annotation and transcriptionally imputed sex label.

GSE21862: McHale et al. (2011) examined PBMC gene expression from 125 Chinese factory workers with different levels of benzene exposure. We included individuals with benzene exposure of less than 1 ppm in our study (“<<1 ppm,” “<1 ppm,” and unexposed controls). We did not detect any mismatched sex labels.

GSE47353: Tsang et al. (2014) profiled PBMC gene expression from 63 healthy volunteers before and after vaccination with the seasonal influenza vaccine. We removed 5 samples due to mismatch between sex annotation and transcriptionally imputed sex

label. We only included samples from the time point prior to vaccination. We used 43 samples from individuals 21-40 years old prior as a discovery cohort. We used 60 samples from individuals 21-62 years old as an exploratory cohort comparing monocyte proportions to Autosomal-iSEXS scores.

GSE53195: Hemani et al. (2014) profiled the whole blood gene expression of individuals from the Brisbane Systems Genetics Study, which included 862 individuals from 374 families. We removed 5 samples due to mismatch between sex annotation and transcriptionally imputed sex label. We included individuals 18-40 years old as a discovery dataset. Due to the age cutoff, only two twin pairs (1 male/female dizygotic, 1 female monozygotic) were included in our analysis.

GSE60491: Vedhara et al. (2015) profiled PBMC gene expression of 119 healthy individuals 18-59 years old in order to find transcriptional differences related to the five major dimensions of human personality (Neuroticism, Extraversion, Openness, Agreeableness, Conscientiousness). We removed one individual due to conflicting sex annotation and transcriptionally imputed sex label. We included individuals 18-40 years old as a discovery dataset.

Validation Cohorts

GSE13485: Querec et al. (2009) profiled PBMC gene expression of 25 healthy people from two different yellow fever vaccine clinical trials. We obtained sex and age annotations in correspondence with the authors. We converted Unigene symbols to gene symbols using the package org.Hs.eg.db (Carlson et al., 2018). We removed 15 samples due to conflicting sex annotation and transcriptionally imputed sex label. We created a validation cohort using samples prior to vaccination from individuals 18-40 years old.

GSE18323: Vahey et al. (2010) profiled PBMC gene expression of 39 individuals aged 18-45 years old vaccinated and challenged with malaria. We used baseline samples (time point 0) as a validation cohort. As age annotation was not available, all individuals were included. As sex annotation was not available, we used transcriptionally imputed sex instead.

GSE19442: Berry et al. (2010) profiled whole blood gene expression of 31 individuals with latent tuberculosis and 20 individuals with active pulmonary tuberculosis. We included individuals with latent tuberculosis 18-40 years old as a validation cohort, because they did not have active infection. No mismatched sex labels were detected.

GSE21311: Mason et al. (2010) profiled whole blood gene expression from 100 healthy individuals, 50 males and 50 females (Mason et al., 2010). We obtained phenotypic annotations and gene expression values in correspondence with the authors, due to widespread incongruence between annotated sex and imputed sex in the publicly available version. We quantile normalized the expression matrix. We did not detect mismatched sex labels. Healthy controls 18-40 years old were used as a validation cohort. We used healthy controls of all ages to examine changes in iSEXS with age.

GSE30453: Heinzen et al. (2008) profiled gene expression from 93 autopsy-collected cortical brain tissue samples and 80 PBMC samples from healthy donors in order to study tissue-specific transcriptomic regulation. We included PBMC samples from individuals 18-40 years old as a validation cohort. We detected no mismatched sex labels.

GSE37069: Seok et al. (2013) profiled gene expression from whole blood of 244 severe burns patients and 35 healthy subjects. We removed 23 samples due to conflicting sex labels and imputed sex. We included healthy individuals 18-40 years old as a validation cohort. We removed sample GSM909661 because it was a duplicate.

GSE38484: de Jong et al. (2012) compared the gene expression from whole blood of 96 healthy controls and 106 individuals with schizophrenia. We removed 9 samples due to conflicting sex labels and imputed sex. Only the healthy controls were included in our analysis. Healthy controls 18-40 years old were used as a validation cohort. Healthy controls of all ages were used to examine changes in iSEXS with age.

GSE58137: Peters et al. (2015) examined transcriptomic changes in whole blood with aging across many cohorts. GSE58137 contains the gene expression of 359 African Americans aged 15-77 years old. The transcriptome was profiled on two different Illumina HumanHT-12 expression beadchip versions: 3.0 and 4.0. We only used samples profiled on version 3.0 because of its larger sample size. We removed 2 samples due to conflicting sex labels and imputed sex.

GSE61821: Hoang et al. (2014) profiled the gene expression in whole blood of individuals infected with influenza. Hoang et al. (2014) included individuals from two different studies: a dengue detection study (EDEN) in Singapore as well as a severe influenza study performed by Southeast Asia Infectious Disease Clinical Research Network (SEAICRN). Only post-infection individuals 18-40 years old from the EDEN study were included in our study as a validation dataset. The SEAICRN study individuals were excluded due to insufficient numbers of individuals 18-40 years old. As sex labels were not available, we used transcriptionally imputed sex.

GSE65219: Kuparinen et al. (2013) examined the gene expression from PBMCs of 146 nonagenarians and 30 younger controls (19-30 years old) from Finland in order to study aging in the immune system. We removed two samples due to conflict between sex annotation and transcriptionally imputed sex. We used the younger controls as a validation cohort.

GSE85263: Rojas-Pena et al. (2018) profiled PBMC gene expression via RNaseq (Illumina HiSeq 2500) in individuals challenged with *Plasmodium vivax* malaria. We used the preprocessed gene transcript counts as provided by the authors. We used the samples collected prior to both malaria vaccination and malaria challenge from individuals 18-40 years old to build a validation cohort. All annotated sex labels matched transcriptionally imputed sex labels.

Exploration Cohorts

GSE42331: Zitzmann et al. (2015) collected whole blood from Klinefelter Syndrome individuals and age-matched male and female controls as part of the EXAKT (Epigenetics X chromosome Features and clinical Applications in Klinefelter Syndrome Trial), a large Munster-based study examining cardiovascular, inflammatory, and metabolic factors in Klinefelter patients (ClinicalTrials.gove

Identifier: NCT01703676) (. We detected no mismatched sex labels, as all XXY-males and XY-males had high expression of RPS4Y1 and KDM5D. We included all individuals in our analysis.

GSE4784: Huang et al. (2015) compared whole blood gene expression between five Klinefelter syndrome (47,XXY) individuals and five age-matched healthy male controls (46,XY). The five Klinefelter syndrome individuals were not undergoing testosterone treatment. The mean ages of Klinefelter syndrome individuals and male controls were 29.8 years and 31.2 years, respectively. The average ages of Klinefelter Syndrome patients, male controls, and female controls were 37.7, 42.4, and 32.6 years old, respectively. We included all individuals in our analysis.

Milieu Interieur Consortium: examines the immune responses of 1000 healthy individuals of French descent 20-69 years old. Piasecka et al. (2018) profiled the expression of 560 immune-related genes via Nanostring in the whole blood of Milieu Interieur Consortium volunteers unstimulated and treated with microbial stimuli. We included 279 unstimulated samples from volunteers 18-40 years old as a validation cohort. Patin et al. (2018) profiled immune cell proportions in the Milieu Interieur cohort via flow cytometry. We included all subjects in our analysis

The Nanostring gene expression data for all subjects is publicly available as supplemental Dataset_S01 of Piasecka et al. (2018). The flow cytometry proportions and subject demographic information is publicly available as the R package mmi (http://github.com/JacobBergstedt/mmi).

GSE65133: Newman et al. (2015) profiled both gene expression and flow cytometry on PBMCs of healthy individuals. Sex labels were not available, so we transcriptionally imputed sex. No age labels were available.

SDY212: Furman et al. (2013) examined whole blood transcriptional responses to the influenza vaccine in 30 younger (20-30 years) and 59 older subjects (60 to >89 years). We obtained the transcriptional and phenotypic data from ImmPort (Bhattacharya et al., 2018). We only used the time point prior to vaccination in our analysis. We removed two samples due to conflict between the sex annotation and the transcriptionally imputed sex.

Fragiadakis et al. & Bjornson et al.: Fragiadakis and Bjornson performed immune cell profiling via mass cytometry in a cohort of 83 healthy volunteers 18-63 years old. Sample preparation and CyTOF methods have been described previously (Fragiadakis et al., 2019). Briefly, peripheral blood was obtained from 86 human adult volunteers from the Stanford Blood Center, AllCells (exempt, non-human subjects research), and the Stanford community under an IRB-approved protocol. Fresh whole blood was fixed and prepared for Cytof analysis. Total monocytes were defined as the summation of classical monocytes (CD11b+ CD16-), non-classical monocytes (CD11b- CD16+), and intermediate monocytes (CD11b+ CD16+). Antibodies were purchased, conjugated using DVS/Fluidigim MaxPar metal conjugation kits, titrated for optimal signal-to-noise ratio, and lyophilized. Data are available upon request.

GSE73072: Liu et al. (2016) examined whole blood gene expression of 2886 samples from 148 individuals from 7 viral challenge studies in order to build individualized infection predictors. Individuals were challenged with influenza, rhinovirus, or respiratory syncytial virus. Each individual's immune response was classified according to two categories: 1) symptomatic versus asymptomatic and 2) viral shedder versus non-shedder. We only included symptomatic shedders from influenza challenges DEE2, DEE3, and DEE5 in our analysis. Influenza challenge DEE4 was excluded because all symptomatic shedders were female. As no sex labels were supplied, sex was imputed transcriptionally. DEE2, DEE3, and DEE5 were processed as described previously (Bongen et al., 2018).

GSE68310: Zhai et al. (2015) profiled the whole blood gene expression of 133 adults with influenza-like illness at the beginning of flu season, during infection, and in the spring after flu season. We removed 7 samples due to mismatch between sex labels and transcriptionally imputed sex. We included individuals with laboratory confirmed influenza in our analysis. No age labels were available.

GSE48018 and GSE48023: Bucasas et al. (2011) profiled whole blood gene expression of 119 male (GSE48018) and 128 female (GSE48023) healthy volunteers 19-41 years old before and after influenza vaccination. We included all samples at the baseline time point in our analysis. All males and females had expected expression patterns of XIST and RPS4Y1.

GSE39088: Lauwerys et al. (2013) profiled the whole blood gene expression of 34 healthy females and 26 SLE patients as part of a clinical trial of the safety of immunization with interferon alpha-Kinoid (IFN-K) (NCT01058343). We included only baseline samples in our analysis. As all samples were female, we did not detect mismatch between sex labels and transcriptionally imputed sex.

GSE49454: Chiche et al. (2014) profiled the whole blood gene expression of 62 SLE patients (53 female, 9 male) and 20 healthy controls (17 female, 3 male). Each SLE patient had 1-6 visit samples available but we only included the earliest sample in our analysis. No sex mislabeled samples were detected.

QUANTIFICATION AND STATISTICAL ANALYSIS

Gene Expression Microarray Dataset Preprocessing

As part of preprocessing, we ensured that each dataset was appropriately normalized and log2-transformed. Unless otherwise noted in the study summary (Supplemental Information), we used all datasets as preprocessed by the original authors. As negative values interfere with the calculation of the geometric mean based iSEXS scores, we raised any gene expression matrix with negative values above zero by adding the absolute value of the minimum value. We ensured that all datasets were log transformed, and performed log-2 transformation if necessary.

Imputing Sex Labels

In datasets where sex annotation was not available, we imputed sex labels using expression of two Y chromosome genes RPS4Y1 and KDM5D as well as XIST, a lncRNA on the X chromosome that is highly expressed in females and mediates X-inactivation (Toker et al., 2016). We performed K-means clustering with two centroids on the expression values of RPS4Y1, KDM5D, and XIST. We assigned the centroid with higher XIST expression and lower RPS4Y1 and KDM5D expression as female. We removed samples from the analysis if their sex label in the GEO did not match their imputed sex label. Of the 21 datasets with available sex labels, 12 contained at least one mislabeled sample with 87 mislabeled samples total (Supplemental Information: Study Summaries). Microarray probes were mapped to Entrez Gene Identifiers (IDs). If a probe mapped to more than one gene, we expanded the expression of that probe to correspond to all relevant genes (Ramasamy et al., 2008).

Integrating Discovery Cohorts by Meta-Analysis

We used MetaIntegrator to apply two meta-analysis methods (combining effect sizes and combining p values) as described previously (Andres-Terre et al., 2015; Bongen et al., 2018; Khatri et al., 2013). We calculated effect sizes as Hedges' adjusted *g* and summarized using random effects inverse variance model, where each effect size was weighted by the inverse of the variance in that study. We corrected the p values for the summary effect size of each gene for multiple hypothesis testing using Benjamini-Hochberg false discovery rate (FDR) (Benjamini and Hochberg, 1995).

We performed meta-analysis by combining p values using Fisher's sum of logs (Fisher, 1934). For each gene, we summed the logarithm of the one-sided hypothesis testing p values across *k* studies and compared the result to a -distribution with 2*k* degrees of freedom.

Statistical Power

Using the method described by Hedges and Pigott, we found our discovery datasets had 90% statistical power at p value of 0.01 for detecting effect size of 0.38, 0.44, 0.54, and 0.78 in the presence of no, low, moderate, or high heterogeneity, respectively (Hedges and Pigott, 2001).

Immune Sex Expression Signature and Score

We arbitrarily defined females as "cases" and males as "controls" so that genes with positive effect sizes were expressed at higher level in females (female-associated iSEXS genes) and genes with negative effect sizes were expressed at a higher level in males (male-associated iSEXS genes). We defined the immune Sex Expression Signature (iSEXS) as differentially expressed genes with an absolute effect size $\geq$ 0.4 and a false discovery rate (FDR) $\leq$ 5% in the discovery cohorts. We defined the iSEXS score of a sample as the geometric mean of female-associated iSEXS gene expression minus the geometric mean of male-associated iSEXS gene expression. We scaled and centered the iSEXS score values within each dataset (mean = 0, standard deviation = 1).

Chromosome Localization

We grouped the iSEXS genes located on sex chromosomes into XY-iSEXS, and those located on autosomes into Autosomal-iSEXS. We identified chromosome location for each gene in iSEXS using the NCBI Entrez Gene annotations from the biomaRt R package Version 2.30 (Durinck et al., 2009). We obtained the X-escape genes from a previously published study by Tukiainen and colleagues (Tukiainen et al., 2017). We used χ^2 test to compute enrichment of X chromosome and Y chromosome genes.

Immune Cell Specificity Scores and Cellular Deconvolution

As described previously, we generated immune cell type specific effect sizes, which are available in the MetaSignature database (http://metasignature.stanford.edu) (Haynes et al., 2017; Vallania et al., 2018). These immune cell type specific effect sizes were generated by combining 6,160 samples from 166 gene expression datasets. For each cell-type-gene pair, we computed the immune cell specificity score as Hedge's g effect sizes of the expression of that gene in that cell type (case) versus all other cell types (controls).

Immune cell type proportions were estimated from bulk blood gene expression using cell mixture deconvolution. We utilized the immunoStates basis matrix and a linear regression model, as described previously (Bongen et al., 2018; Roy Chowdhury et al., 2018; Vallania et al., 2018).

Statistical Tests

We used t tests to compare the distributions of two groups. To calculate significance of the interactions between age and sex we used two-way Anova with an unbalanced design using Type-III sums of squares. To convert age into a factor individuals were binned into two groups: younger (18-40 years old) and older (50+ years old).

DATA AND CODE AVAILABILITY

All datasets used here are publicly available with access described in Supplemental Information (Data S1). The code used for analysis is available at GitHub (https://github.com/ebongen/iSEXS_cellReports2019).

Cell Reports, Volume *29*

Supplemental Information

Sex Differences in the Blood Transcriptome Identify Robust Changes in Immune Cell Proportions with Aging and Influenza Infection

Erika Bongen, Haley Lucian, Avani Khatri, Gabriela K. Fragiadakis, Zachary B. Bjornson, Garry P. Nolan, Paul J. Utz, and Purvesh Khatri

Supplemental Information

Contents

Fig. S1. Performance of XY- and Autosomal-iSEXS scores in discovery cohorts

Fig. S2. XXY-males express iSEXS scores and genes similarly to females

Fig. S3. Autosomal-iSEXS score reflects age-dependent sex differences in monocyte percentages.

Fig. S4. XY-iSEXS score does not change over time in females or males following influenza challenge

Fig. S5. iSEXS and Antibody Responses

Fig. S6. XY-iSEXS and Autosomal-iSEXS in SLE datasets

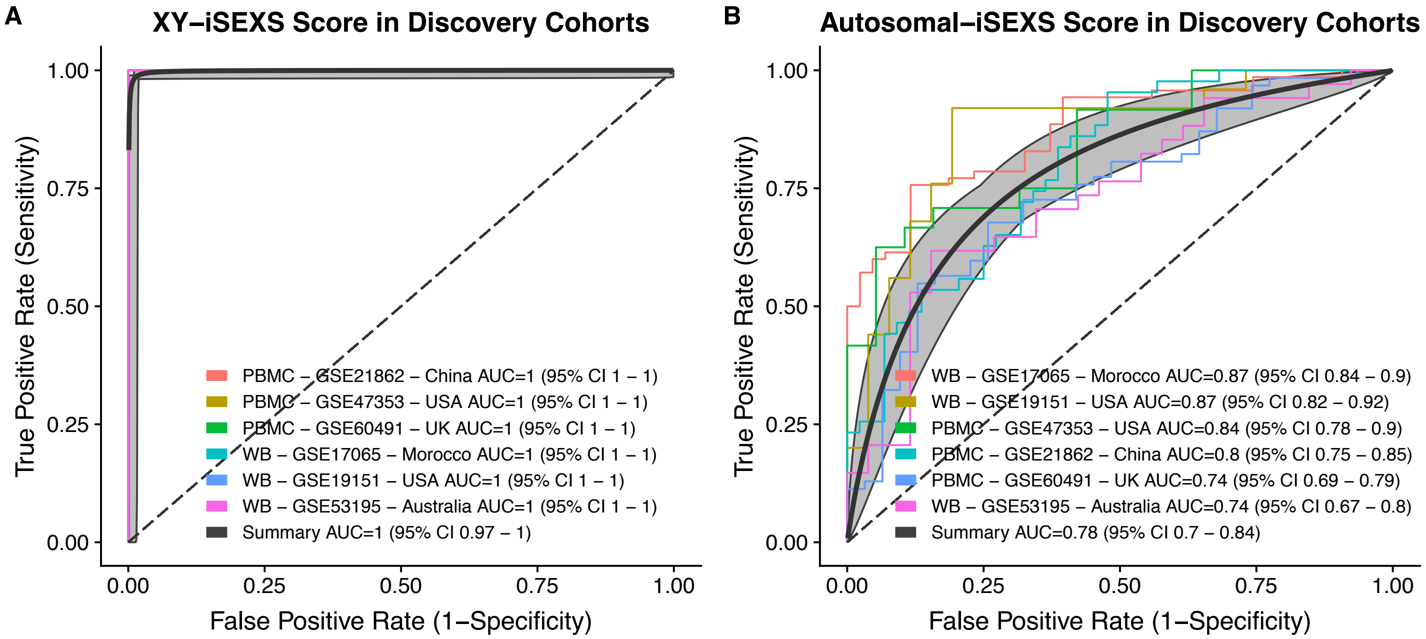

Figure S1. Performance of XY- and Autosomal-iSEXS scores in discovery cohorts. Related to Figure 2. ROC plots of the A) XY-iSEXS score (summary AUROC=1.00 95% CI 0.97-1.00) and B) Autosomal-iSEXS score (summary AUROC=0.78, 95% CI 0.7-0.84) performance in separating males and females 18-40 years old in the discovery cohorts.

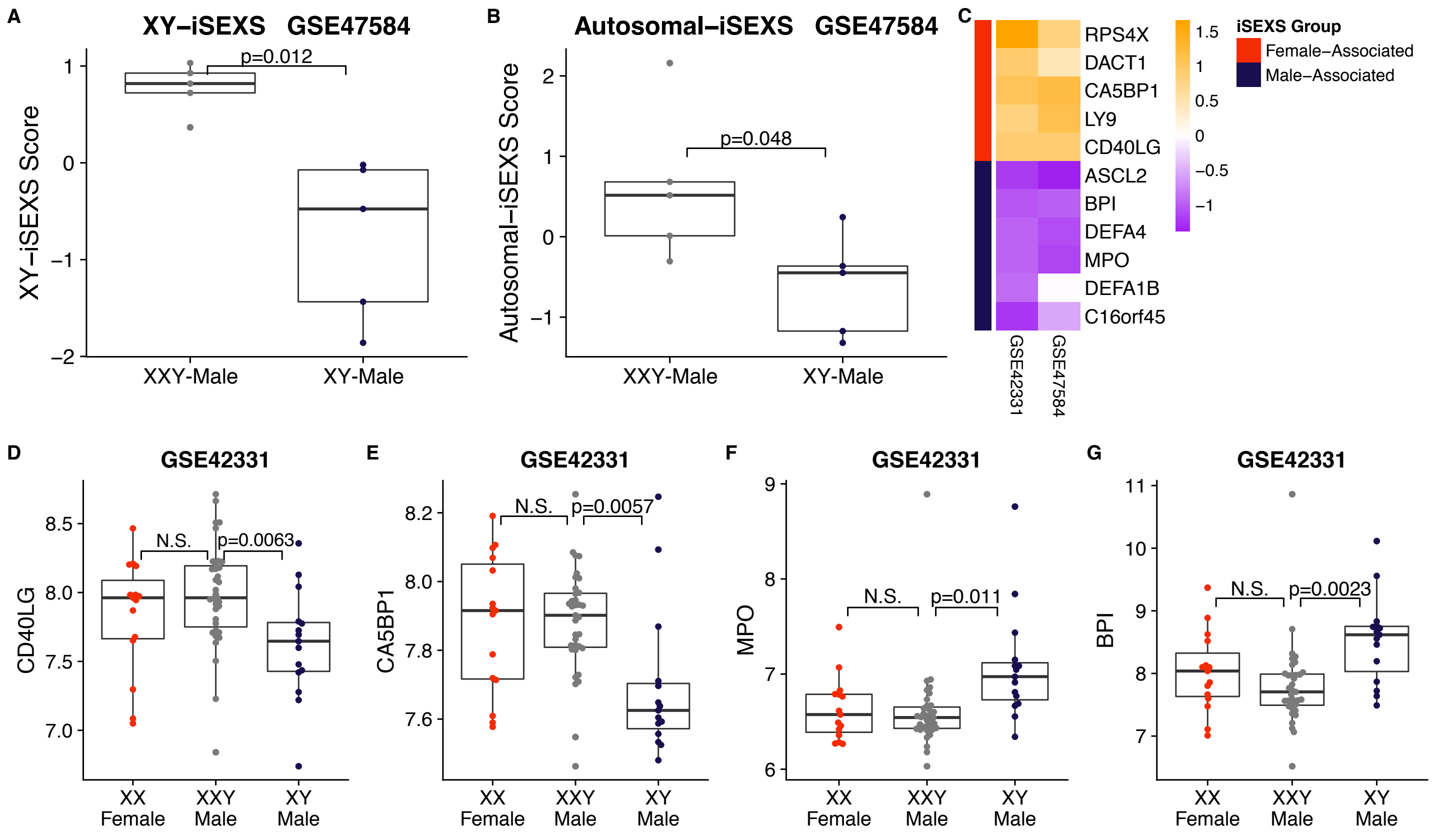

Figure S2. XXY-males express iSEXS scores and genes similarly to females. Related to Figure 2. Boxplots of A) XY-iSEXS and B) Autosomal-iSEXS scores in Klinefelter males (XXY-males) and typical males (XY-males). C) Heatmap of iSEXS genes significantly different between XXY-males and XY-males in a meta-analysis of GSE42331 and GSE47584 (FDR < 5%). Orange indicates genes higher expressed in XXY-males, purple indicates genes higher expressed in XY-males. Row color key indicates iSEXS genes higher expressed in females (red) or males (blue). Box plots of genes differentially expressed between XXY- and XY-males in cohort GSE42331: D) *CD40LG*, E) *CA5BP1*, F) *MPO*, and G) *BPI*.

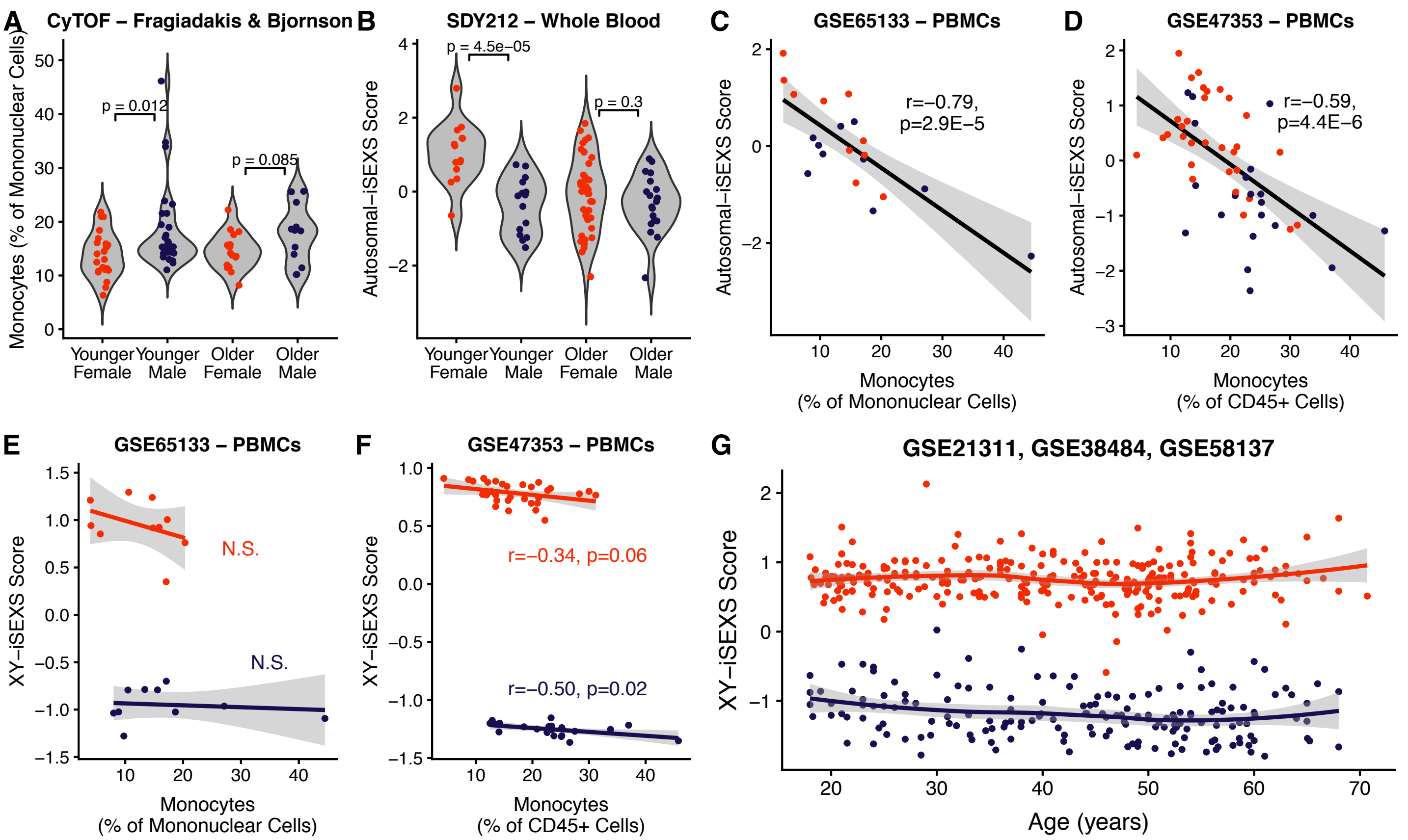

Figure S3. Autosomal-iSEXS score reflects age-dependent sex differences in monocyte percentages. Related to Figure 4. A) Among younger individuals (18-40 years old), males have a higher percentage of monocytes in the blood, as measured by CyTOF (p=0.012), but this difference is not significant in older individuals (41-63 years old; p=0.085). B) In SDY212, the Autosomal-iSEXS score is significantly higher in younger females (20-30 years old; p=4.5E-5), but demonstrates no significant difference in older individuals (60-90 years old; p=0.30). Autosomal-iSEXS scores correlate with flow cytometry measured monocyte percentages in C) GSE65133 (r=-0.79, p=2.9E-05) and D) GSE47353 (r=-0.59, p=4.4E-06). E) XY-iSEXS scores do not correlate with monocyte percentages GSE65133, (F) but significantly correlate in males in GSE47353 (r=-0.50, p=0.02) with a non-significant trend in females (r=-0.34, p=0.06). G) The XY-iSEXS scores are consistent across the life course in the combined cohorts of GSE21311, GSE38484, and GSE58137.

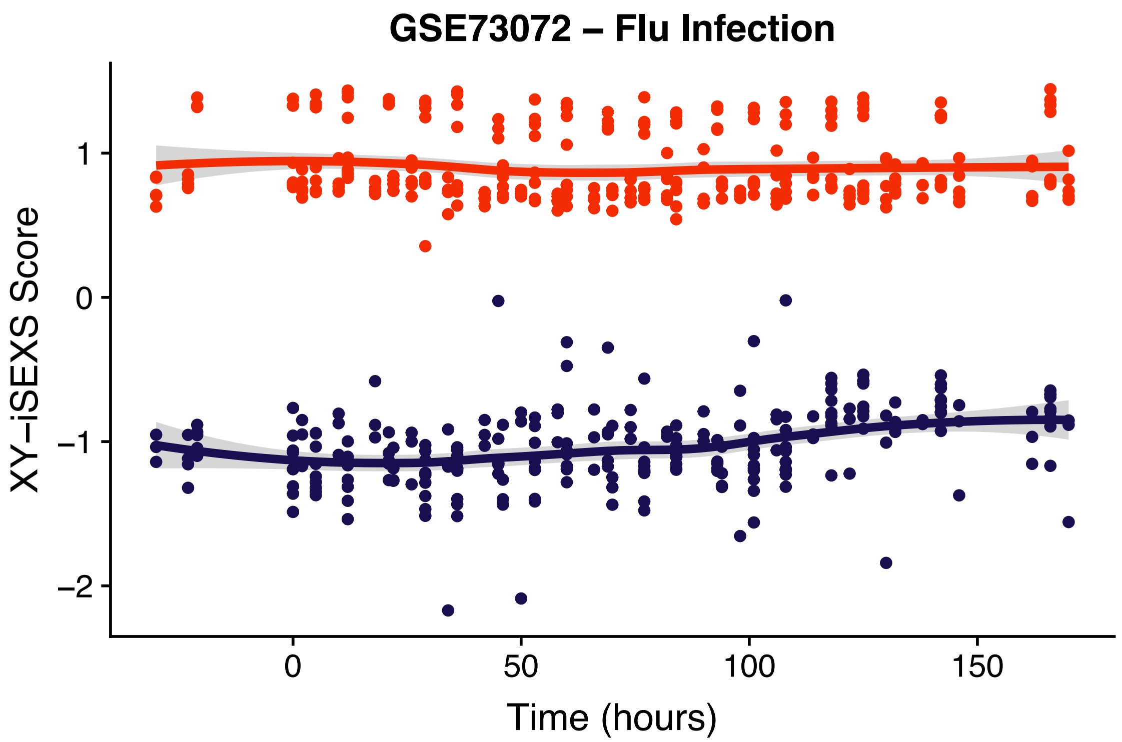

Figure S4. XY-iSEXS score does not change over time in females or males following influenza challenge. Related to Figure 5.

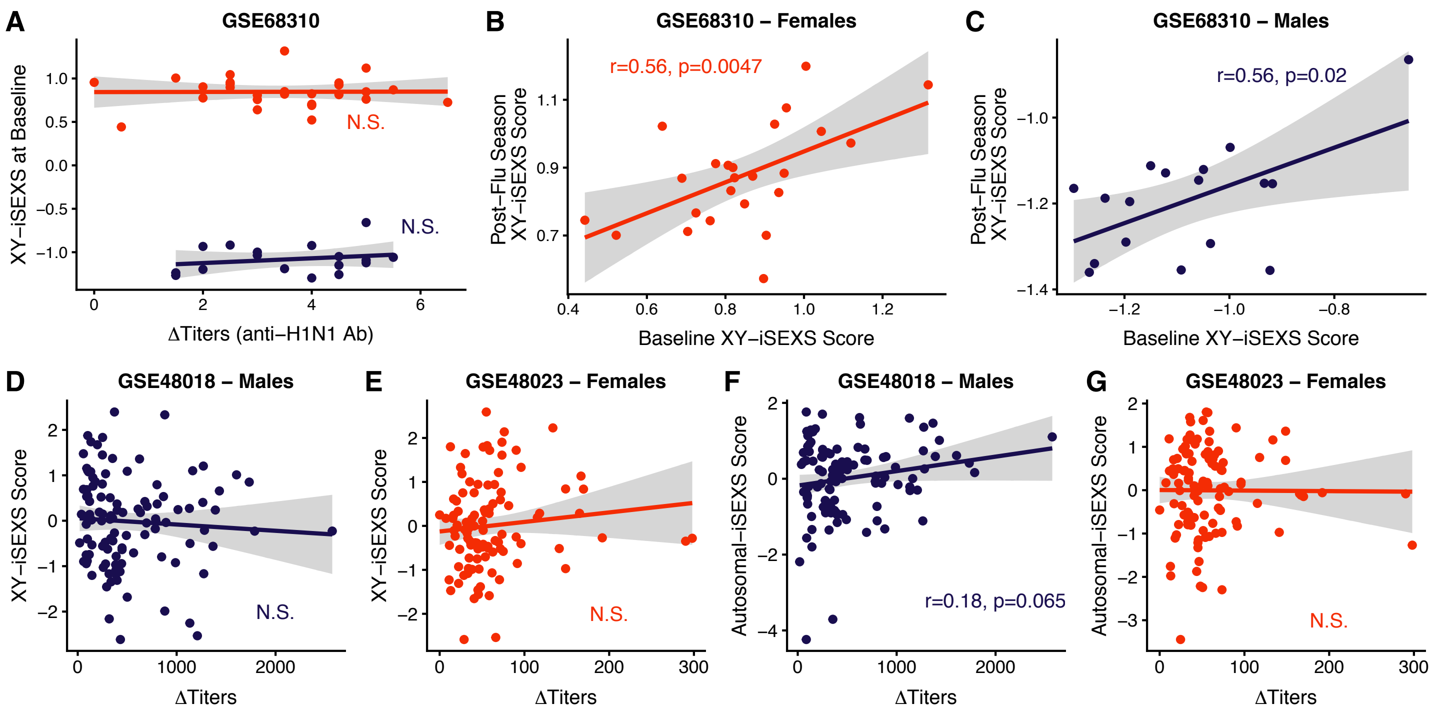

Figure S5. **iSEXS and Antibody Responses. Related to Figure 6.** A) XY-iSEXS at baseline is not correlated with change in titer post-influenza infection. B-C) XY-iSEXS score pre- and post-influenza infection is significantly correlated in females and males. Baseline D-E) XY-iSEXS score does not significantly correlate with antibody response to influenza vaccination. Correlation between baseline Autosomal-iSEXS score and ΔTiters in F) males (GSE48018; r=0.18, p=0.065) and G) females (GSE48023; r=-0.0063, p=0.95). In GSE48018 and GSE48023, antibody titers were calculated as the geometric mean of the antibody titers against the three influenza strains tested, and ΔTiters was the difference between the geometric mean titer at day 28 and day 0.

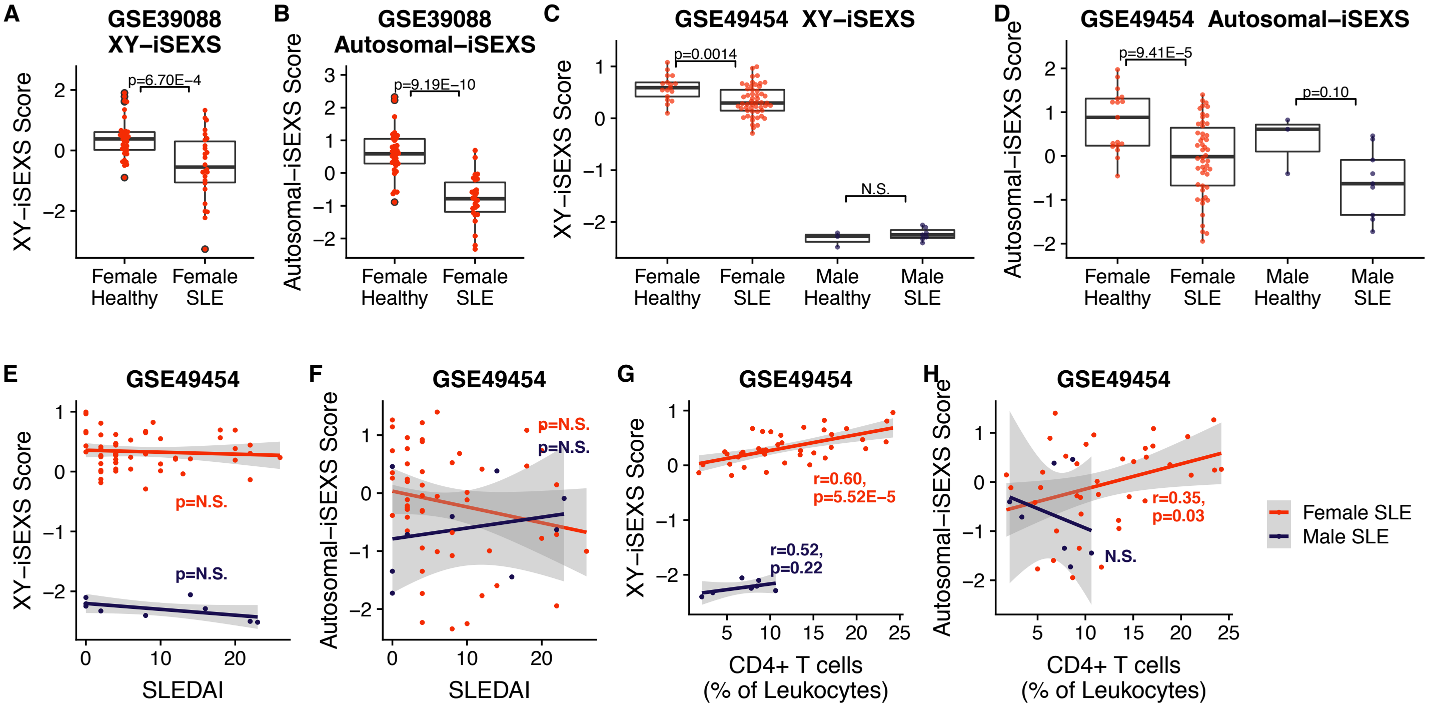

Fig. S6. XY-iSEXS and Autosomal-iSEXS in SLE datasets. Related to Figure 6. (A-D) XY-iSEXS and Autosomal-iSEXS are lower in females with SLE compared to healthy females. In males, Autosomal-iSEXS is significantly lower in males with SLE compared to healthy males, but XY-iSEXS is not. **(E-F)** XY-iSEXS is inversely correlated with SLEDAI in males but not in females. Autosomal-iSEXS is not correlated with SLEDAI in either females or males with SLE. **(G-H)** XY-iSEXS and Autosomal-iSEXS are positively correlated with proportion of CD4+ T cells in females, whereas only XY-iSEXS is negatively correlated in males. Autosomal-iSEXS is not correlated with proportions of CD4+ T cells in males.
